# Supplementary material for: Kernel Architecture of the Genetic Circuitry of the Arabidopsis Circadian System
Source: PLoS Comput Biol. 2016 Feb 1;12(2):e1004748. doi: 10.1371/journal.pcbi.1004748 (PMC4734688; doi:10.1371/journal.pcbi.1004748)
Supplement: S1 Text — (PDF) [file pcbi.1004748.s001.pdf]

Kernel Architecture of the Genetic Circuitry of the *Arabidopsis* Circadian System

Mathias Foo, David E. Somers, Pan-Jun Kim

S1 Text

Table of contents

S1 Text. Extended methods and results

S1. System identification of the *Arabidopsis* circadian system: MF2015 3

S1.1. Data collection 3

S1.2. Model structure selection 5

S1.3. Comparison with previous models 8

S1.4. Parameter estimation 9

S1.5. Model validation 10

S1.6. Sensitivity analysis 11

S2. Kernel of the *Arabidopsis* circadian system 12

S2.1. Kernel identification 12

S2.2. Robustness of the kernel compared with MF2015 16

S3. Negative feedback loops in the kernel structure 16

S3.1. Characterization of loops I to IV 16

S3.2. Regulatory effect of PRR proteins 20

S3.3. Inhibition of *PRR5* by LHY/CCA1 20

S4. Prevalent inhibitory interactions in the *Arabidopsis* circadian system 21

S4.1. Relationship between cuspidate profiles and regulatory interactions 21

S4.2. Revisiting *PRR5* inhibition by LHY/CCA1 23

S1–S49 References 25

S1. System identification of the *Arabidopsis* circadian system: MF2015

System identification techniques were pioneered by Lennart Ljung [1], and have been widely used for the construction of mathematical models [2, 3]. There are four main steps involved in system identification: data collection, model structure selection, parameter estimation and model validation. In this study, we followed these steps to construct our model (MF2015) of the core circadian clock in *Arabidopsis thaliana*. Several models for plant circadian clocks based on system identification have been published [4, 5]; however, these linear black-box models do not facilitate biological interpretations, and they considered only specific subsections of the plant circadian system.

S1.1. Data collection

Light is the major external signal for entrainment of the plant circadian system. Five different light conditions are typically implemented in laboratories to study the effect of light on the plant circadian system: 12 hours of light and 12 hours of dark (12L:12D cycle), 16 hours of light and 8 hours of dark (long day), 8 hours of light and 16 hours of dark (short day), constant light (LL), and constant dark (DD).

Time-course expression data for 24 mRNAs and proteins of wild-type (WT) *Arabidopsis* under different light conditions were used for our model parameter estimation. All data were obtained from publicly available literature and databases. The sources of mRNA expression data were usually quantitative polymerase chain reaction (qPCR) analyses, whereas those for protein expression data were immunoblots (Western blots).

The absolute levels of mRNAs and proteins are difficult to ascertain from the common literature and databases; therefore, the expression levels were normalized to dimensionless values (≤ 1) with arbitrary scales. If the original data have amplitudes scaled between 0 and 1, we used the data without additional processing. If the data have amplitudes > 1, we normalized the data by their maximum amplitude. For cases where mRNA or protein expression levels were presented in image formats, especially for proteins, we transformed the images into quantifiable numbers using digitizing software and normalized them with respect to the maximum amplitude value. Our specific normalization method is not of concern from a parameter-estimation viewpoint, because the absolute amplitude scales are absorbed into the model parameters. We stress that our model is meaningful for a relative change in the expression levels of a given

component (mRNA, protein, or protein complex), but it is not meaningful for a comparison between absolute expression levels of different components.

S1 Table summarizes the data sources for mRNA and protein levels used in our study. As a proxy for *LATE ELONGATED HYPOCOTYL* (*LHY*) and *CIRCADIAN CLOCK ASSOCIATED 1* (*CCA1*) expression, we adopted the data for *LHY* mRNA and LHY protein levels. Although *LHY* and *CCA1*, technically, can be considered individually, they have very similar behaviors and are often estimated as a single element [6-9]. Moreover, data quality for *LHY* often was better than that for *CCA1* in the literature sources. Therefore, we adopted *LHY* data as a proxy for *LHY* and *CCA1* data, and the model outputs for *LHY* mRNA and LHY protein represent the joint activity of *LHY* and *CCA1*.

There are no available experimental profiles for EVENING COMPLEX (EC), protein P, and CONSTITUTIVE PHOTOMORPHOGENESIS PROTEIN 1 (COP1). Therefore, we utilized partially elucidated information for these components. EC is formed by EARLY FLOWERING 3 (ELF3), EARLY FLOWERING 4 (ELF4), and LUX ARRHYTHMO (LUX) [10]. Based on the profiles of these three components, we presumed that the EC levels would peak at zeitgeber time (ZT) 16 under 12L:12D cycles, and estimated the profile as a sinusoidal wave with the same peak time. Protein P accumulates in darkness and is degraded in light [6]. Accordingly, we estimated the profile of protein P as exponentially rising and falling in darkness and in light, respectively. We estimated the profile of COP1 as similar to that of protein P based on the information in refs. [8, 11, 12].

Typically, these time-course data points have 4-hour sampling intervals. The available data with higher time resolution (sampling interval < 4 hours) indicate that mRNA and protein expression levels behave continuously over time [13]; therefore, we interpolated the data points to have 1-hour intervals. Different interpolation methods gave similar results in the model parameter estimation, and we present the linear interpolation results here. To estimate the model parameters and account for sustained oscillations in WT plants, the first-day data were replicated for the next three days. Data from LL or DD experiments show damped oscillations in expression levels over days. To estimate the time-invariant, inherent oscillation in WT, we only considered expression profiles in the first circadian cycles [24.6 hours for LL [14] and 25.9 hours for DD [15]] and replicated them for subsequent cycles. In this way, consecutive datasets of five different light conditions were developed for each mRNA or protein in the following order:

12L:12D cycles, short days, long days, LL, and DD. Each constant condition (LL or DD) was preceded by the entrainment under a 12L:12D cycle. If experimental values were not available for a particular light condition, we filled that missing part with null data [16], which are not actually used for any calculation in this study. Finally, to consider experimental errors in the measured data or to require minimal levels of system robustness to molecular fluctuation, we added slight random fluctuations only to initial concentrations for each light condition, and thereby generated six additional time-course datasets for every case.

S1.2. Model structure selection

There are two types of models in system identification: a black-box model or a gray-box model. A black-box model is a set of general model structures that describe an abstract relation between input and output and does not provide mechanistic insights into the system. A gray-box model uses mechanistic knowledge about the system when selecting an appropriate model structure [17]. We adopted a gray-box model for MF2015 based on ordinary differential equations (ODEs) that describe transcriptional, translational, and post-translational events.

Most ODEs in MF2015 follow the forms of

$$\dot{c}^m(t) = f_1[\{c^{TF}(t)\},\{h\},\{\theta\}] - g_1[c^m(t),\{\theta\}]$$

and

$$\dot{c}^p(t) = f_2[c^m(t),\{\theta\}] - g_2[c^p(t),\{\theta\}] ,$$

where c^m (c^p) denotes mRNA (protein) concentration, c^{TF} denotes the transcription factor concentration, t is time, the function f_1 (f_2) describes transcriptional (translational) mechanisms, the function g_1 (g_2) describes mRNA (protein) degradation, θ's are model parameters, h's are the Hill coefficients, and $\{\cdots\}$ includes single or multiple elements. If experimental evidence indicates that transcription factors form a dimer, we set the Hill coefficient to 2; otherwise, it is set to 1 [8, 18].

Transcriptional regulation was modeled by Michaelis-Menten kinetics, with $\theta_1(c^{TF})^h/[\theta_2^h + (c^{TF})^h]$ for activation and $\theta_1/[\theta_2^h + (c^{TF})^h]$ for inhibition. The regulatory effect of multiple activators (inhibitors) was modeled by the summation (product) of individual regulatory effects. When PSEUDO RESPONSE REGULATORs 9, 7, 5 (PRR9, PRR7, and PRR5), and TIMING OF CAB EXPRESSION 1 (TOC1) regulate the same gene, we considered a weighted sum of their concentrations instead of the individual concentrations separately, because these

proteins are homologous and likely to bind to similar regulatory sites [13, 14, 19, 20]. The unweighted sum of the concentrations can be used if we know their absolute concentrations; however, we do not know that information, so we used the weighted sum. We modeled the binding of ZEITLUPE (ZTL) and GIGANTEA (GI) by adapting the alternative Michaelis-Menten relation suggested in [21]. We took the weighted sum of ZTL and GI concentrations for the denominator in this relation, because we do not know their absolute concentrations, which would allow the unweighted sum. GI stabilizes ZTL and slows its degradation; therefore, the binding of ZTL and GI, in effect, contributes positive terms to a rate of the net ZTL level change in our model.

Accordingly, MF2015 consists of the following ODEs (Fig 1A):

$$\frac{dc_P(t)}{dt} = \theta_1 L(t) + \theta_2 c_P(t) + \theta_3 \quad \text{[S1]}$$

$$\frac{dc_L^m(t)}{dt} = \theta_4 c_P(t)L(t) + \frac{\theta_5}{\theta_6^2 + \theta_7[\theta_{78}c_{P9}^p(t) + \theta_{79}c_{P7}^p(t) + \theta_{80}c_{P5}^p(t) + c_T^p(t)]^2} + \theta_8 c_L^m(t) \quad \text{[S2]}$$

$$\frac{dc_L^p(t)}{dt} = \theta_{13}c_L^m(t) + \theta_{14}c_L^p(t) \quad \text{[S3]}$$

$$\begin{aligned}\frac{dc_{P9}^m(t)}{dt} &= \theta_{15}c_P(t)L(t) + \frac{\theta_{16}}{[\theta_{17} + c_{EC}^p(t)]\{\theta_{18}^2 + \theta_{19}[\theta_{80}c_{P5}^p(t) + c_T^p(t)]^2\}} \\ &\quad + \frac{\theta_{20}c_L^p(t)^2}{\theta_{21}^2 + c_L^p(t)^2} + \theta_{22}c_{P9}^m(t)\end{aligned} \quad \text{[S4]}$$

$$\frac{dc_{P9}^p(t)}{dt} = \theta_{27}c_{P9}^m(t) + \theta_{28}c_{P9}^p(t) \quad \text{[S5]}$$

$$\begin{aligned}\frac{dc_{P7}^m(t)}{dt} &= \frac{\theta_{29}}{[\theta_{30} + c_{EC}^p(t)]\{\theta_{31}^2 + \theta_{32}[\theta_{80}c_{P5}^p(t) + c_T^p(t)]^2\}} + \frac{\theta_{33}c_L^p(t)^2}{\theta_{34}^2 + c_L^p(t)^2} \\ &\quad + \theta_{35}c_{P7}^m(t)\end{aligned} \quad \text{[S6]}$$

$$\frac{dc_{P7}^p(t)}{dt} = \theta_{40}c_{P7}^m(t) + \theta_{41}c_{P7}^p(t) \quad \text{[S7]}$$

$$\frac{dc_{P5}^m(t)}{dt} = \frac{\theta_{43}}{[\theta_{44}^2 + c_T^p(t)^2][\theta_{45}^2 + c_L^p(t)^2]} + \frac{\theta_{46}c_{R8}^p(t)^2}{\theta_{47}^2 + c_{R8}^p(t)^2} + \theta_{48}c_{P5}^m(t) \quad \text{[S8]}$$

$$\frac{dc_{P5}^p(t)}{dt} = \theta_{53}c_{P5}^m(t) + \frac{\theta_{54}c_{ZTL}^p(t)c_{GI}^p(t)c_{P5}^p(t)}{1 + \theta_{144}c_{ZTL}^p(t) + \theta_{145}c_{GI}^p(t)} + \theta_{55}c_{P5}^p(t) \quad \text{[S9]}$$

$$\frac{dc_T^m(t)}{dt} = \frac{\theta_{60}}{[\theta_{61} + c_{EC}^p(t)][c_{62}^2 + c_L^p(t)^2]} + \frac{\theta_{63} c_{R8}^p(t)^2}{\theta_{64}^2 + c_{R8}^p(t)^2} + \theta_{65} c_T^m(t) \quad \text{[S10]}$$

$$\frac{dc_T^p(t)}{dt} = \theta_{70} c_T^m(t) + \frac{\theta_{71} c_{ZTL}^p(t) c_{GI}^p(t) c_T^p(t)}{1 + \theta_{144} c_{ZTL}^p(t) + \theta_{145} c_{GI}^p(t)} + \theta_{72} c_T^p(t) \quad \text{[S11]}$$

$$\frac{dc_{EC}^p(t)}{dt} = \theta_{74} c_{E3}^p(t) c_{E4}^p(t) c_{LUX}^p(t) + \theta_{75} c_{EC}^p(t) \quad \text{[S12]}$$

$$\frac{dc_{R8}^m(t)}{dt} = \frac{\theta_{76}}{\theta_{77}^2 + [\theta_{78} c_{P9}^p(t) + \theta_{79} c_{P7}^p(t) + \theta_{80} c_{P5}^p(t)]^2} + \theta_{81} c_{R8}^m(t) \quad \text{[S13]}$$

$$\frac{dc_{R8}^p(t)}{dt} = \theta_{85} c_{R8}^m(t) + \theta_{86} c_{R8}^p(t) \quad \text{[S14]}$$

$$\frac{dc_{E3}^m(t)}{dt} = \frac{\theta_{87}}{\theta_{88}^2 + c_L^P(t)^2} + \theta_{89} c_{E3}^m(t) \quad \text{[S15]}$$

$$\frac{dc_{E3}^p(t)}{dt} = \theta_{93} c_{E3}^m(t) + \theta_{94} c_{CP}^p(t) c_{E3}^p(t) + \theta_{95} c_{E3}^p(t) \quad \text{[S16]}$$

$$\frac{dc_{E4}^m(t)}{dt} = \frac{\theta_{99}}{[\theta_{100} + c_{EC}^p(t)][\theta_{101}^2 + c_L^p(t)^2]} + \frac{\theta_{102} c_{R8}^p(t)^2}{\theta_{103}^2 + c_{R8}^p(t)^2} + \theta_{104} c_{E4}^m(t) \quad \text{[S17]}$$

$$\frac{dc_{E4}^p(t)}{dt} = \theta_{109} c_{E4}^m(t) + \theta_{110} c_{E4}^p(t) \quad \text{[S18]}$$

$$\frac{dc_{LUX}^m(t)}{dt} = \frac{\theta_{112}}{[\theta_{113} + c_{EC}^p(t)][\theta_{114}^2 + c_L^p(t)^2]} + \frac{\theta_{115} c_{R8}^p(t)^2}{\theta_{116}^2 + c_{R8}^p(t)^2} + \theta_{117} c_{LUX}^m(t) \quad \text{[S19]}$$

$$\frac{dc_{LUX}^p(t)}{dt} = \theta_{122} c_{LUX}^m(t) + \theta_{123} c_{LUX}^p(t) \quad \text{[S20]}$$

$$\frac{dc_{GI}^m(t)}{dt} = \theta_{125} c_P(t) L(t) + \frac{\theta_{126}}{[\theta_{127}^2 + c_L^p(t)^2][\theta_{128}^2 + c_T^p(t)^2][\theta_{129}^2 + c_{E3}^p(t)^2]} + \theta_{130} c_{GI}^m(t) \quad \text{[S21]}$$

$$\frac{dc_{GI}^p(t)}{dt} = \theta_{135} c_{GI}^m(t) + \theta_{136} c_{CP}^p(t) c_{E3}^p(t) c_{GI}^p(t) + \theta_{137} c_{GI}^p(t) \quad \text{[S22]}$$

$$\frac{dc_{CP}^p(t)}{dt} = \theta_{140} L(t) + \theta_{141} c_{CP}^p(t) + \theta_{142} \quad \text{[S23]}$$

$$\frac{dc_{ZTL}^p(t)}{dt} = \frac{\theta_{143} L(t) c_{ZTL}^p(t) c_{GI}^p(t)}{1 + \theta_{144} c_{ZTL}^p(t) + \theta_{145} c_{GI}^p(t)} + \frac{\theta_{146} c_{ZTL}^p(t) c_{GI}^p(t)}{1 + \theta_{144} c_{ZTL}^p(t) + \theta_{145} c_{GI}^p(t)} + \theta_{147} c_{ZTL}^p(t) + \theta_{148} \quad \text{[S24]}$$

where $L(t)$ denotes a light intensity as a function of time, either 1 (light) or 0 (dark) when simulating the aforementioned five different light conditions. c^m (c^p) corresponds to mRNA (protein or protein complex) concentration, and has a dimensionless value with arbitrary scale for each component (mentioned previously with respect to normalization); therefore, values within the same component, but not between different components, can be meaningfully compared. Subscripts '*P*', '*L*', '*P9*', '*P7*', '*P5*', '*T*', '*EC*', '*R8*', '*E3*', '*E4*', '*LUX*', '*GI*', '*CP*', and '*ZTL*' denote protein P, *LHY* (as a proxy for *LHY/CCA1*), *PRR9*, *PRR7*, *PRR5*, *TOC1*, EC, *REVEILLE 8* (*RVE8*), *ELF3*, *ELF4*, *LUX*, *GI*, COP1, and ZTL, respectively. θ's are model parameters.

In Eq. (S1)–(S24), there are some jumps in the indices of θ's. For example, the last parameter in Eq. (S2) is θ_8, which is then followed by θ_{13} in Eq. (S3). The omitted indices are for scaling factors for c^m's and c^p's across different light conditions; the experimental data do not show the absolute expression levels for each component in different light conditions, and have been normalized by us as discussed previously. Hence, there is no necessity that c^m and c^p from different conditions match all the normalized experimental data in their amplitudes, although the waveforms themselves should be matched. If we multiply c^m or c^p by a proper coefficient (scaling factor) in each condition, then this scaled c^m or c^p can match the normalized experimental amplitude. Therefore, the inverses of the scaling factors roughly estimate the relative levels of actual amplitudes across different light conditions. Without loss of generality, we set the scaling factors for 12L:12D cycles to be 1, and introduce the scaling factors for other light conditions where the experimental data for a given component are available. The full list of parameter values along with scaling factors is presented in S2 Table (dimensionless values; time in hours for the model simulation). The detailed procedures to obtain those parameters and scaling factors are described later.

S1.3. Comparison with previous models

Our new model is mainly based on the model (P2013) by Pokhilko et al. [9], but we filtered out hypothetical or outdated molecular interactions and adopted some recent findings [22]. For example, we removed from P2013 sequential activation of the PRR wave, and added the transcriptional activator RVE8 [23] in a fashion similar to the model (F2014) by Fogelmark and Troein [22]. The degradation of the EC by GI [8] was not considered in our model due to a lack of direct evidence. We also removed the inhibition of the EC genes by TOC1, because it can, at

least in part, be explained by the net effect of a triple-negative connection that TOC1 represses *PRR* gene expression, PRR proteins repress *LHY/CCA1* expression, and LHY/CCA1 represses the EC gene expression. Unlike F2014, our model retains *LHY/CCA1* as a single component rather than splits them into *LHY* and *CCA1*. This policy still allowed good model performance, without introducing additional complexity. The transcription factor in F2014, NADPH oxidases/BROTHER OF LUX ARRHYTHMO (NOX/BOA), was not considered in our model, as we expect that NOX/BOA and LUX would be similar in their behaviors, and thus the function of NOX/BOA may be represented by that of LUX. Our model maintains mathematically consistent forms of kinetic functions throughout most equations, to support the reader's clear and intuitive interpretation. Additionally, compared to our earlier work [16], which uses a discrete-time model for control design purposes, here we have constructed a continuous-time model, with revised interactions compatible with recent knowledge.

S1.4. Parameter estimation

The datasets described in Section S1.1 were used for estimation of parameters [Eq. (S1)–(S24)] and scaling factors in our model. The parameters were estimated by minimizing the prediction error function [the right-hand side of Eq. (S25)] [1], using the MATLAB function *fminsearch*.

$$\{\hat{\theta}\} = \arg\min_{\{\theta\}} \sum_i \frac{1}{N_i} \sum_{t=1}^{N_i} [c_{i,e}^{\alpha_i}(t) - \theta_{i,E(t)}^{s} \cdot c_{i,m}^{\alpha_i}(t,\{\theta\})]^2 \qquad \text{[S25]}$$

where i denotes each of the 24 molecular components in Eq. (S1)–(S24), and α indicates mRNA, protein, or protein complex. $c_{i,e}$ and $c_{i,m}$ are experimental and simulated molecular concentrations, respectively (the ODEs were solved using the MATLAB function *ode45*). t denotes time (with 1-hour intervals), spanning different light conditions and initial conditions (Section S1.1). θ^s is a scaling factor specific for each component i and light condition E. N is the number of available time points for each component. $\hat{\theta}$'s are the estimated parameters including scaling factors. In cases where constraints need to be imposed on the parameter values to avoid over-fitting or biologically unrealistic solutions, the MATLAB function *fmincon,* instead of *fminsearch,* was used for the parameter optimization. The initial parameters to start the optimization were chosen using a linear least square method described in [16]; it assumes a linear equation for each mRNA or protein production, and presumably results in the initial parameters not extremely far from the

optima. S2 Table lists the final estimated parameters. All estimated parameters are dimensionless (time in hours for the model simulation) because the experimental data were normalized.

Based on these estimated parameters, S1–S5 Fig compares the simulated data with experimental data. Overall, the model outputs resemble the experimental data. There are several instances where the model does not closely match the experimental data. Most of these are associated with amplitude mismatch. Because the experimental data do not provide information for absolute amplitude levels, we are not currently much concerned about those mismatches. There are also phase mismatch cases, although no phase mismatch is larger than 4 hours. Because the typical experimental data have 4-hour sampling intervals, the observed phase mismatches appear to be within an acceptable range. In contrast, under 12L:12D cycles, P2013 shows 5- and 10-hour phase mismatches in TOC1 and ZTL profiles, respectively.

The oscillation periods under constant conditions, simulated by our model, are in good agreement with experimental values [14, 15]: 25.2 h (model) versus 24.6 h (experiment) in LL, and 25.8 h (model) versus 25.9 h (experiment) in DD. Our model predicts the ZTL protein levels over time (S6 Fig), which qualitatively match the experimental report [24]. The experiment reported the substantial elevation (reduction) of ZTL levels in LL (DD). To our knowledge, this is the first computational demonstration of these ZTL behaviors.

S1.5. Model validation

In the context of system identification, the term *model validation* is used to evaluate the capability of the model to reproduce the dynamics of the system when model outputs are compared with new datasets that have not been used for parameter estimation. For this purpose, we simulated various mutants in MF2015 and compared their free running periodicity with experimental data (S3 Table). When simulating a gene mutation, we reduced the transcription rate to half of its original value; when simulating gene overexpression, we added a constant to the transcription rate. For every simulation, we initially entrained the mutant under two 12L:12D cycles, and subsequently transferred the simulation condition to LL or DD. In LL or DD, the Fourier transform of each mRNA or protein profile gave rise to the respective period, and the periods were averaged over the components that did not exhibit arrhythmia. If all the components were arrhythmic, we classified the clock outcome as arrhythmic.

S3 Table presents the experimental and simulation results. If those two results exhibit ≤ 4-hour differences in their periods or both show arrhythmias, we consider them to match quantitatively (because many experimental data were obtained with 4-hour sampling intervals). Such quantitative agreement was observed for 76.2% of the simulation outcomes and experimental results. Qualitative agreement (lengthened period, shortened period, or arrhythmia) was observed for 85.7% of the simulation outcomes and experimental results. Most of the mismatches occurred in DD. This is expected considering the limited amount of experimental data in DD, which could be accessed during model construction. For generality, we also simulated each gene mutation by reducing the transcription rate to arbitrary values. Simulated *Δprr7* in LL (with a ~10-to-40%-reduced transcription rate) and in DD (with a ~20-to-40%-reduced transcription rate), and *Δelf3* in DD (with an up-to-20%-reduced transcription rate) provide additional examples of quantitative agreement with experimental mutant periods. We stress that MF2015 does not aim to outperform existing models [9, 22] in terms of its accuracy for mutant data, but intends to relevantly work for our kernel identification for WT plants.

S1.6. Sensitivity analysis

We analyzed the sensitivity of the model to changes in parameters and initial conditions. Sensitivity was quantified by the root mean square error (RMSE) as follows:

$$\mathrm{RMSE} = \left\{ \frac{1}{N} \sum_{t=1}^{N} [c_f(t) - c_o(t)]^2 \right\}^{1/2} \quad \text{[S26]}$$

where c_f and c_o are simulated expression levels of a particular component under a particular light condition (the system was entrained in two 12L:12D cycles before transferred to the light condition of interest); c_f is a simulated value when we perturb each parameter or initial condition, whereas c_o is the original simulated value without those perturbations. After each parameter or initial condition perturbation, we identified the largest RMSE ($\mathrm{RMSE_{max}}$) among all components and light conditions, except for GI and ZTL in LL ($\mathrm{RMSE_{GI,LL}}$ and $\mathrm{RMSE_{ZTL,LL}}$). Based on our manual inspection, if the model outcome simultaneously satisfies $\mathrm{RMSE_{max}} \leq 0.2$, $\mathrm{RMSE_{GI,LL}} \leq 0.5$, and $\mathrm{RMSE_{ZTL,LL}} \leq 0.5$, it can be considered as robust to the perturbation (because GI and ZTL levels are substantially elevated in LL, they allow relatively large RMSEs).

For the parameter sensitivity analysis, we multiplied each parameter by a factor K_P ($0 \leq K_P \leq 2$) with increments of 0.1 (i.e., a total of 21 K_P values), while holding the other parameters

constant. For this parameter, we then counted the number of K_P values (# K_P) that satisfy $RMSE_{max} \le 0.2$, $RMSE_{GI,LL} \le 0.5$, and $RMSE_{ZTL,LL} \le 0.5$. The larger # K_P, the more robust the model is to this parameter perturbation. We found that most parameters have # $K_P > 3$ (e.g., 70 parameters have # $K_P = 21$). Only six parameters showed # $K_P \le 3$, and they were involved in *PRR5* mRNA degradation, *ELF3* inhibition by LHY/CCA1, and the production of protein P and COP1 protein. To examine the effect of altered parameters on the predictability of mutant period lengths (Section S1.5), we multiplied the parameters (with # $K_P = 21$) by random values of K_P between 0 and 2, and then generated five different parameter sets giving rise to the WT expression profiles. These alternative parameter sets show similar results to the previous mutant predictability. For example, from one parameter set among the five, observed were only two meaningful improvements in the predictability: 28.8 h (LL) and 29.8 h (DD) for the *Δprr7* mutant additionally match the experimental values (S3 Table) within ±4 h.

For the initial condition sensitivity analysis, we varied from 0 to 2 (with an increment of 0.1) the initial concentration of each component under each light condition, while holding those of the other components constant. For every perturbed initial condition, we found $RMSE_{max} \le 0.2$, $RMSE_{GI,LL} \le 0.5$, and $RMSE_{ZTL,LL} \le 0.5$.

Together, these results suggest that the overall simulation outcomes are robust to changes in parameters and initial conditions, i.e., transient molecular concentrations.

S2. Kernel of the *Arabidopsis* circadian system

In this study, we define a kernel as a collection of spanning subgraphs that satisfy the following condition: each spanning subgraph contains all molecular components in the system and a minimal subset of their regulatory interactions (including light regulation), which are necessary to generate the temporal trajectory of molecular concentrations close to those of WT.

S2.1. Kernel identification

Ideally, our kernel identification procedure would be to search through all possible combinations of regulatory interactions (including light regulation), and examine the effects when those regulations in each combination are removed with parameter re-optimization to best fit the original model outputs across different light conditions. This strategy, although ideal, is extremely computationally demanding. Instead, we devised a heuristic approach as follows (in

this procedure, both molecular interactions and light regulations are referred to simply as interactions): first, we simulated the knockout effect of each interaction on WT expression patterns under five different light conditions. The knockout effects were quantified by RMSE, similar to Eq. (S26). The only difference is that here we define c_f as the simulated data under the removal of a given interaction. After deletion of a given interaction, we calculated RMSEs for all components and light conditions, and identified the largest value ($RMSE_{max}$) among these RMSEs except for GI and ZTL proteins in LL ($RMSE_{GI,LL}$ and $RMSE_{ZTL,LL}$). Based on our manual inspection, the model outputs appear to remain robust if they simultaneously satisfy $RMSE_{max} \leq 0.2$, $RMSE_{GI,LL} \leq 0.5$, and $RMSE_{ZTL,LL} \leq 0.5$ (because GI and ZTL levels are substantially elevated in LL, they allow relatively large RMSEs). From MF2015, we pruned all 16 interactions with small knockout effects ($RMSE_{max} \leq 0.2$, $RMSE_{GI,LL} \leq 0.5$, and $RMSE_{ZTL,LL} \leq 0.5$). The simulated profiles with the only remaining interactions after the pruning still showed $RMSE_{max} \leq 0.2$, $RMSE_{GI,LL} \leq 0.5$, and $RMSE_{ZTL,LL} \leq 0.5$. Among these remaining interactions, we focused on the five interactions that satisfy $RMSE_{max} \leq 0.3$, $RMSE_{GI,LL} \leq 0.8$, and $RMSE_{ZTL,LL} \leq 0.8$. We found that two of the five can be additionally removed from the system because the simultaneous deletion of both of these interactions eventually resulted in $RMSE_{max} \leq 0.2$, $RMSE_{GI,LL} \leq 0.5$, and $RMSE_{ZTL,LL} \leq 0.5$, when parameter re-optimization was performed (at this time, we re-optimized the parameters in a fashion similar to Section S1.4, but without considering the randomly-disturbed initial conditions). The deletion of any other interaction among the remaining three failed to result in such small RMSEs, even when parameters were re-optimized. For the other interactions with larger $RMSE_{max}$, $RMSE_{GI,LL}$, or $RMSE_{ZTL,LL}$ ($RMSE_{max} > 0.3$, $RMSE_{GI,LL} > 0.8$, or $RMSE_{ZTL,LL} > 0.8$) with the original parameters, we did not attempt to remove them because these RMSEs usually did not fall below the thresholds of $RMSE_{max} = 0.2$, $RMSE_{GI,LL} = 0.5$, and $RMSE_{ZTL,LL} = 0.5$ when parameter re-optimization was performed. Conceptually, our method may appear similar to previous studies pruning neural networks [25, 26].

We leave some remarks for the above procedures during kernel identification. The *PRR7* inhibition by TOC1 was initially removed because of its small RMSEs. In fact, the small RMSEs resulted from the *PRR7* inhibition by the EC, which buffered the loss of the inhibition by TOC1. The profiles of TOC1 and EC are similar in MF2015; therefore, the loss of the *PRR7* inhibition by the EC was compensated for by the inhibition by TOC1 with parameter re-optimization.

Because the two inhibitory relationships are equivalent, we reinstate the *PRR7* inhibition by TOC1 in the kernel structure. Another remark is that the scaling factors for different light conditions, and θ_{144} and θ_{145} for the binding of GI and ZTL proteins (Section S1.2), were not changed much during parameter re-optimization; therefore, we held them constant as the original values (this also reduces the computational time for our parameter re-optimization). Technically, those scaling factors and particular parameters harbor information of the absolute concentrations of the corresponding components, and thus are favored to be maintained as constant.

The dynamics of the resulting kernel structure with 22 regulatory interactions and light regulations (Fig 1A) is described as the following equations:

$$\frac{dc_P(t)}{dt} = \phi_1 L(t) + \phi_2 c_P(t) + \phi_3 \quad [S27]$$

$$\frac{dc_L^m(t)}{dt} = \frac{\phi_4}{\phi_5^2 + \phi_6[\phi_7 c_{P9}^p(t) + \phi_8 c_{P7}^p(t) + \phi_9 c_{P5}^p(t)]^2} + \phi_{10} c_L^m(t) \quad [S28]$$

$$\frac{dc_L^p(t)}{dt} = \phi_{11} c_L^m(t) + \phi_{12} c_L^p(t) \quad [S29]$$

$$\frac{dc_{P9}^m(t)}{dt} = \phi_{13} c_P(t) L(t) + \phi_{14} + \frac{\phi_{15} c_L^p(t)^2}{\phi_{16}^2 + c_L^p(t)^2} + \phi_{17} c_{P9}^m(t) \quad [S30]$$

$$\frac{dc_{P9}^p(t)}{dt} = \phi_{18} c_{P9}^m(t) + \phi_{19} c_{P9}^p(t) \quad [S31]$$

$$\frac{dc_{P7}^m(t)}{dt} = \frac{\phi_{20}}{[\phi_{21} + c_{EC}^p(t)][\phi_{22}^2 + c_T^p(t)^2]} + \phi_{23} c_{P7}^m(t) \quad [S32]$$

$$\frac{dc_{P7}^p(t)}{dt} = \phi_{24} c_{P7}^m(t) + \phi_{25} c_{P7}^p(t) \quad [S33]$$

$$\frac{dc_{P5}^m(t)}{dt} = \frac{\phi_{26}}{[\phi_{27}^2 + c_T^p(t)^2][\phi_{28}^2 + c_L^p(t)^2]} + \phi_{29} c_{P5}^m(t) \quad [S34]$$

$$\frac{dc_{P5}^p(t)}{dt} = \phi_{30} c_{P5}^m(t) + \phi_{31} c_{P5}^p(t) \quad [S35]$$

$$\frac{dc_T^m(t)}{dt} = \frac{\phi_{32}}{[\phi_{33} + c_{EC}^p(t)][\phi_{34}^2 + c_L^p(t)^2]} + \phi_{35} c_T^m(t) \quad [S36]$$

$$\frac{dc_T^p(t)}{dt} = \phi_{36} c_T^m(t) + \phi_{37} c_T^p(t) \quad [S37]$$

$$\frac{dc_{EC}^{p}(t)}{dt} = \phi_{38} c_{E3}^{p}(t) c_{E4}^{p}(t) c_{LUX}^{p}(t) + \phi_{39} c_{EC}^{p}(t) \quad [S38]$$

$$\frac{dc_{R8}^{m}(t)}{dt} = \frac{\phi_{40}}{\phi_{41}^{2}(t) + \phi_{9} c_{P5}^{p}(t)^{2}} + \phi_{42} c_{R8}^{m}(t) \quad [S39]$$

$$\frac{dc_{R8}^{p}(t)}{dt} = \phi_{43} c_{R8}^{m}(t) + \phi_{44} c_{R8}^{p}(t) \quad [S40]$$

$$\frac{dc_{E3}^{m}(t)}{dt} = \frac{\phi_{45}}{\phi_{46}^{2} + c_{L}^{p}(t)^{2}} + \phi_{47} c_{E3}^{m}(t) \quad [S41]$$

$$\frac{dc_{E3}^{p}(t)}{dt} = \phi_{48} c_{E3}^{m}(t) + \phi_{49} c_{E3}^{p}(t) \quad [S42]$$

$$\frac{dc_{E4}^{m}(t)}{dt} = \frac{\phi_{50}}{[\phi_{51} + c_{EC}^{p}(t)][\phi_{52}^{2} + c_{L}^{p}(t)^{2}]} + \phi_{53} c_{E4}^{m}(t) \quad [S43]$$

$$\frac{dc_{E4}^{p}(t)}{dt} = \phi_{54} c_{E4}^{m}(t) + \phi_{55} c_{E4}^{p}(t) \quad [S44]$$

$$\frac{dc_{LUX}^{m}(t)}{dt} = \frac{\phi_{56}}{[\phi_{57} + c_{EC}^{p}(t)][\phi_{58}^{2} + c_{L}^{p}(t)^{2}]} + \phi_{59} c_{LUX}^{m}(t) \quad [S45]$$

$$\frac{dc_{LUX}^{p}(t)}{dt} = \phi_{60} c_{LUX}^{m}(t) + \phi_{61} c_{LUX}^{p}(t) \quad [S46]$$

$$\frac{dc_{GI}^{m}(t)}{dt} = \frac{\phi_{62}}{[\phi_{63}^{2} + c_{L}^{p}(t)^{2}][\phi_{64}^{2} + c_{T}^{p}(t)^{2}][\phi_{65}^{2} + c_{E3}^{p}(t)^{2}]} + \phi_{66} c_{GI}^{m}(t) \quad [S47]$$

$$\frac{dc_{GI}^{p}(t)}{dt} = \phi_{67} c_{GI}^{m}(t) + \phi_{68} c_{CP}^{p}(t) c_{E3}^{p}(t) c_{GI}^{p}(t) + \phi_{69} c_{GI}^{p}(t) \quad [S48]$$

$$\frac{dc_{CP}^{p}(t)}{dt} = \phi_{70} L(t) + \phi_{71} c_{CP}^{p}(t) + \phi_{72} \quad [S49]$$

$$\frac{dc_{ZTL}^{p}(t)}{dt} = \frac{\phi_{73} c_{ZTL}^{p}(t) c_{GI}^{p}(t)}{1 + \theta_{144} c_{ZTL}^{p}(t) + \theta_{145} c_{GI}^{p}(t)} + \phi_{74} c_{ZTL}^{p}(t) + \phi_{75} \quad [S50]$$

where ϕ's are parameters of the kernel (S4 Table). These parameters were estimated in a fashion similar to Section S1.4, but without considering the randomly-disturbed initial conditions. For the cases where experimental profiles are not available for particular components and light conditions, we used the MF2015-simulated profiles as their substitutes during the parameter

estimation. S1–S5 Fig suggests that the WT expression profiles simulated from the kernel structure [Eq. (S27)–(S50)] match the overall experimental and MF2015-simulated profiles.

S2.2. Robustness of the kernel compared with MF2015

We compared the kernel and MF2015 in terms of their robustness to perturbations of parameters and initial conditions. For either the kernel or MF2015 under a particular perturbation, we obtained RMSEs using Eq. (S26). In a similar way to Section S1.6, if $\mathrm{RMSE}_{\mathrm{max}} \leq 0.2$, $\mathrm{RMSE}_{\mathrm{GI,LL}} \leq 0.5$, and $\mathrm{RMSE}_{\mathrm{ZTL,LL}} \leq 0.5$, we consider the system as robust to the perturbation.

First, for each parameter, we varied K_P (in Section S1.6) from 0 to 2 under a given light condition, and obtained the range of K_P (ΔK_P) where the system remains robust. We found that MF2015 tends to have larger ΔK_P than the kernel. Across all parameter perturbations and light conditions, MF2015 has relatively large ΔK_P 1.4 times more often than the kernel. Next, for each component, we varied the initial concentration from 0 to 2 under a given light condition, and obtained the range of the initial concentration (Δc) where the system remains robust. We found that the kernel never exceeds MF2015 in its Δc across all initial condition perturbations and light conditions. To summarize, MF2015 tends to be more robust than the kernel, indicating that the extra interactions in MF2015, which are not present in the kernel, may improve the robustness of the plant circadian system when subjected to perturbations.

S3. Negative feedback loops in the kernel structure

The kernel structure embeds four major negative feedback loops, designated as loops I to IV (Fig 2). Here, we investigate the dynamical capability of loops I to IV.

S3.1. Characterization of loops I to IV

Loops I to IV contain the following components: loop I contains *LHY/CCA1*, *PRR5*, and *TOC1* (Fig 2A). Loop II contains *LHY/CCA1*, *PRR7*, and *TOC1* (Fig 2B). Loop III contains *LHY/CCA1*, *PRR7*, and the EC, along with the EC subcomponents (Fig 2C). Loop IV contains *LHY/CCA1*, and *PRR9* regulated by light (Fig 2D). Loop I is not structurally identical, but similar to a repressilator; loops II and III are structurally identical to a repressilator [27].

We first checked how much each loop can contribute to the system's endogenous (autonomous or free running) oscillation by examining the loop's oscillatory capability to imitate

WT expression patterns under constant conditions. Specifically, from MF2015, we extracted the equations for each loop and its linear upstream pathway in the kernel (i.e., light signaling through PRR9 in the cases of loops I to III), and re-optimized the parameters to fit the WT expression profiles of these components in LL. The parameter re-optimization was conducted in a similar way to Section S2.1 where we estimated the parameters of the kernel structure. The linear upstream pathway appended to each loop is considered here to simply reflect the light intensity effect of LL on the simulation results, and does not interfere with the essential characteristics of the loop's intrinsic dynamics under such a constant condition.

The equations for loops I to IV coupled with their linear upstream pathway in the kernel are given by the following equations:

Loop-I case

$$\frac{dc_P(t)}{dt} = \alpha_1 L(t) + \alpha_2 c_P(t) + \alpha_3 \quad \text{[S51]}$$

$$\frac{dc_L^m(t)}{dt} = \frac{\alpha_4}{\alpha_5^2 + \alpha_6[\theta_{78} c_{P9}^p(t) + \theta_{80} c_{P5}^p(t)]^2} + \alpha_7 c_L^m(t) \quad \text{[S52]}$$

$$\frac{dc_L^p(t)}{dt} = \alpha_8 c_L^m(t) + \alpha_9 c_L^p(t) \quad \text{[S53]}$$

$$\frac{dc_{P9}^m(t)}{dt} = \alpha_{10} c_P(t) L(t) + \alpha_{11} c_{P9}^m(t) \quad \text{[S54]}$$

$$\frac{dc_{P9}^p(t)}{dt} = \alpha_{12} c_{P9}^m(t) + \alpha_{13} c_{P9}^p(t) \quad \text{[S55]}$$

$$\frac{dc_{P5}^m(t)}{dt} = \frac{\alpha_{14}}{[\alpha_{15}^2 + c_T^p(t)^2][\alpha_{16}^2 + c_L^p(t)^2]} + \alpha_{17} c_{P5}^m(t) \quad \text{[S56]}$$

$$\frac{dc_{P5}^p(t)}{dt} = \alpha_{18} c_{P5}^m(t) + \alpha_{19} c_{P5}^p(t) \quad \text{[S57]}$$

$$\frac{dc_T^m(t)}{dt} = \frac{\alpha_{20}}{\alpha_{21}^2 + c_L^p(t)^2} + \alpha_{22} c_T^m(t) \quad \text{[S58]}$$

$$\frac{dc_T^p(t)}{dt} = \alpha_{23} c_T^m(t) + \alpha_{24} c_T^p(t) \quad \text{[S59]}$$

Loop-II case

$$\frac{dc_P(t)}{dt} = \beta_1 L(t) + \beta_2 c_P(t) + \beta_3 \quad \text{[S60]}$$

$$\frac{dc_L^m(t)}{dt} = \frac{\beta_4}{\beta_5^2 + \beta_6[\theta_{78} c_{P9}^p(t) + \theta_{79} c_{P7}^p(t)]^2} + \beta_7 c_L^m(t) \quad \text{[S61]}$$

$$\frac{dc_L^p(t)}{dt} = \beta_8 c_L^m(t) + \beta_9 c_L^p(t) \quad \text{[S62]}$$

$$\frac{dc_{P9}^m(t)}{dt} = \beta_{10} c_P(t) L(t) + \beta_{11} c_{P9}^m(t) \quad \text{[S63]}$$

$$\frac{dc_{P9}^p(t)}{dt} = \beta_{12} c_{P9}^m(t) + \beta_{13} c_{P9}^p(t) \quad \text{[S64]}$$

$$\frac{dc_{P7}^m(t)}{dt} = \frac{\beta_{14}}{\beta_{15}^2 + c_T^p(t)^2} + \beta_{16} c_{P7}^m(t) \quad \text{[S65]}$$

$$\frac{dc_{P7}^p(t)}{dt} = \beta_{17} c_{P7}^m(t) + \beta_{18} c_{P7}^p(t) \quad \text{[S66]}$$

$$\frac{dc_T^m(t)}{dt} = \frac{\beta_{19}}{\beta_{20}^2 + c_L^p(t)^2} + \beta_{21} c_T^m(t) \quad \text{[S67]}$$

$$\frac{dc_T^p(t)}{dt} = \beta_{22} c_T^m(t) + \beta_{23} c_T^p(t) \quad \text{[S68]}$$

Loop-III case

$$\frac{dc_P(t)}{dt} = \gamma_1 L(t) + \gamma_2 c_P(t) + \gamma_3 \quad \text{[S69]}$$

$$\frac{dc_L^m(t)}{dt} = \frac{\gamma_4}{\gamma_5^2 + \gamma_6[\theta_{78} c_{P9}^p(t) + \theta_{79} c_{P7}^p(t)]^2} + \gamma_7 c_L^m(t) \quad \text{[S70]}$$

$$\frac{dc_L^p(t)}{dt} = \gamma_8 c_L^m(t) + \gamma_9 c_L^p(t) \quad \text{[S71]}$$

$$\frac{dc_{P9}^m(t)}{dt} = \gamma_{10} c_P(t) L(t) + \gamma_{11} c_{P9}^m(t) \quad \text{[S72]}$$

$$\frac{dc_{P9}^p(t)}{dt} = \gamma_{12} c_{P9}^m(t) + \gamma_{13} c_{P9}^p(t) \quad \text{[S73]}$$

$$\frac{dc_{P7}^{m}(t)}{dt} = \frac{\gamma_{14}}{\gamma_{15} + c_{EC}^{P}(t)} + \gamma_{16} c_{P7}^{m}(t) \quad \text{[S74]}$$

$$\frac{dc_{P7}^{p}(t)}{dt} = \gamma_{17} c_{P7}^{m}(t) + \gamma_{18} c_{P7}^{p}(t) \quad \text{[S75]}$$

$$\frac{dc_{EC}^{p}(t)}{dt} = \gamma_{19} c_{E3}^{p}(t) c_{E4}^{p}(t) c_{LUX}^{p}(t) + \gamma_{20} c_{EC}^{p}(t) \quad \text{[S76]}$$

$$\frac{dc_{E3}^{m}(t)}{dt} = \frac{\gamma_{21}}{\gamma_{22}^{2} + c_{L}^{p}(t)^{2}} + \gamma_{23} c_{E3}^{m}(t) \quad \text{[S77]}$$

$$\frac{dc_{E3}^{p}(t)}{dt} = \gamma_{24} c_{E3}^{m}(t) + \gamma_{25} c_{E3}^{p}(t) \quad \text{[S78]}$$

$$\frac{dc_{E4}^{m}(t)}{dt} = \frac{\gamma_{26}}{[\gamma_{27} + c_{EC}^{P}(t)][\gamma_{28}^{2} + c_{L}^{P}(t)^{2}]} + \gamma_{29} c_{E4}^{m}(t) \quad \text{[S79]}$$

$$\frac{dc_{E4}^{p}(t)}{dt} = \gamma_{30} c_{E4}^{m}(t) + \gamma_{31} c_{E4}^{p}(t) \quad \text{[S80]}$$

$$\frac{dc_{LUX}^{m}(t)}{dt} = \frac{\gamma_{32}}{[\gamma_{33} + c_{EC}^{p}(t)][\gamma_{34}^{2} + c_{L}^{p}(t)^{2}]} + \gamma_{35} c_{LUX}^{m}(t) \quad \text{[S81]}$$

$$\frac{dc_{LUX}^{p}(t)}{dt} = \gamma_{36} c_{LUX}^{m}(t) + \gamma_{37} c_{LUX}^{p}(t) \quad \text{[S82]}$$

Loop-IV case

$$\frac{dc_{P}(t)}{dt} = \eta_{1} L(t) + \eta_{2} c_{P}(t) + \eta_{3} \quad \text{[S83]}$$

$$\frac{dc_{L}^{m}(t)}{dt} = \frac{\eta_{4}}{\eta_{5}^{2} + \eta_{6}\theta_{78} c_{P9}^{p}(t)^{2}} + \eta_{7} c_{L}^{m}(t) \quad \text{[S84]}$$

$$\frac{dc_{L}^{p}(t)}{dt} = \eta_{8} c_{L}^{m}(t) + \eta_{9} c_{L}^{p}(t) \quad \text{[S85]}$$

$$\frac{dc_{P9}^{m}(t)}{dt} = \eta_{10} c_{P}(t) L(t) + \frac{\eta_{11} c_{L}^{p}(t)^{2}}{\eta_{12}^{2} + c_{L}^{p}(t)^{2}} + \eta_{13} c_{P9}^{m}(t) \quad \text{[S86]}$$

$$\frac{dc_{P9}^{p}(t)}{dt} = \eta_{14} c_{P9}^{m}(t) + \eta_{15} c_{P9}^{p}(t) \quad \text{[S87]}$$

where α's, β's, γ's, and η's are estimated parameters (S5 Table) as described above. Fig 3A–3C shows that loops I, II, and III can maintain endogenous oscillations close to those of WT, whereas loop IV cannot. We therefore suggest that, among the four loops, loop IV would not have the strongest role in the formation of the system's endogenous oscillation, as further clarified below.

S3.2. Regulatory effect of PRR proteins

The parameters θ_{78}, θ_{79}, and θ_{80} in Eq. (S1)–(S24) quantify the relative strength of inhibitory regulation by PRR9, PRR7, and PRR5, respectively (as indicated in Section S1.2, θ_{78}, θ_{79}, and θ_{80} also harbor information on the absolute concentrations of these PRR proteins).

In S2 Table for MF2015, θ_{78} is 3.4 and 23.7 times smaller than θ_{79} and θ_{80}, respectively. If we remove the *LHY/CCA1* inhibition by PRR5 or PRR7 from MF2015 [by setting $c_{P5}^{p}(t)$ or $c_{P7}^{p}(t)$ to zero only in Eq. (S2)], it inactivates loop I or both loops II and III, respectively. These simulated mutants largely restore their oscillatory profiles in LL after parameter re-optimization, and reveal the complementary relationships between the loops (Fig 3D). When conducting these types of loop perturbations, combined with parameter re-optimization in LL, we observe that θ_{78} is consistently smaller (~2.6 to 13.2 times smaller) than either θ_{79} or θ_{80}. In other words, through our simulation, the *LHY/CCA1* inhibition by PRR9 (in loop IV) is consistently weaker than either the *LHY/CCA1* inhibition by PRR7 or that by PRR5 (in loops I to III). This result naturally comes from a relatively weak role of loop IV in the formation of the circadian oscillation, as discussed above. Experimental evidence in Fig 3E supports our suggestion.

S3.3. Inhibition of *PRR5* by LHY/CCA1

Loop I includes *PRR5* inhibition by LHY/CCA1, placed in opposition to the repressilator's overall cyclic direction; by default, this interaction might retard the loop's inherent oscillation. In fact, this retardation effect does not only affect the loop-I dynamics but also affects the whole MF2015 dynamics, because of the structural interconnection between loop I and the whole system. For example, MF2015 simulation demonstrates that a 20% increase in the *PRR5* inhibition by LHY/CCA1 [by substituting $1.2\,c_{L}^{p}(t)^{2}$ for $c_{L}^{p}(t)^{2}$ in Eq. (S8)] lengthens the clock period from 25.2 to 28.5 hours in LL, whereas a 20% decrease of this inhibition [by substituting $0.8\,c_{L}^{p}(t)^{2}$ for $c_{L}^{p}(t)^{2}$ in Eq. (S8)] shortens the period from 25.2 to 22.3 hours in LL (Fig 3F). In

contrast to MF2015, P2013 assumes that LHY/CCA1 activates *PRR5* expression. P2013 simulation of LL shows that an increase or decrease in the *PRR5* activation by LHY/CCA1 barely changes the clock period, until an 80% decrease of the activation lengthens the period from 23.4 to 24.3 hours.

S4. Prevalent inhibitory interactions in the *Arabidopsis* circadian system

The kernel structure, as well as the whole MF2015, is overwhelmingly composed of inhibitory interactions between genes. Because the kernel structure is designed for the production of temporal gene expression patterns close to WT, we presumed that many inhibitory regulations, at least in the kernel, may be associated with specific waveforms of the WT expression profiles. Indeed, a number of *Arabidopsis* clock genes often exhibit *cuspidate* waveforms of mRNA and protein expression over time (Fig 4B and S1–S5 Fig). We explored the relationship between cuspidate waveforms and inhibitory/activating interactions.

S4.1. Relationship between cuspidate profiles and regulatory interactions

To investigate the formation of cuspidate profiles, we consider a mathematical system consisting of a single transcription factor (either inhibitor or activator) and its own target gene. The equations for this system are given by

$$\frac{dx_m(t)}{dt} = g[x_{TF}(t), h, \{\alpha\}] - \lambda_m x_m(t) \qquad \text{[S88]}$$

$$\frac{dx_p(t)}{dt} = x_m(t) - \lambda_p x_p(t) \qquad \text{[S89]}$$

where x_{TF} denotes the transcription factor concentration, x_m (x_p) denotes the target gene's mRNA (protein) concentration, t is time, $g = x_{TF}(t)^h/[\alpha_1+\alpha_2 \cdot x_{TF}(t)^h] + \alpha_3$ if the transcription factor is an activator, $g = 1/[\alpha_1+\alpha_2 \cdot x_{TF}(t)^h]$ if the transcription factor is an inhibitor, h is the Hill coefficient, and α's and λ's are constants. In Eq. (S89), without loss of generality, we omitted the coefficient for a protein synthesis rate per mRNA in front of $x_m(t)$. Therefore, technically, $x_m(t)$ should be interpreted as the protein synthesis rate, rather than as the mRNA concentration itself. In a similar way to some equations in MF2015, we set $h = 2$ unless specified, and $h = 1$ did not change the main results. We set $\lambda_p \leq \lambda_m$, regarding that proteins tend to be degraded more slowly. Although Eq. (S88) was formulated for the case of a single transcription factor, it functions for

multiple transcription factors as well, because the combined activity profile of these transcription factors [represented by g in Eq. (S88)] can be mapped into a mathematically equivalent single transcription factor's profile.

What form of $x_{TF}(t)$ renders $x_p(t)$ close to a cuspidate waveform in Fig 4B? To answer this question, we considered various $x_{TF}(t)$ waveforms and whether the transcription factor is an activator or inhibitor. For each case, we time-shifted the $x_{TF}(t)$ waveform, hour by hour, and considered every possible peak time of $x_{TF}(t)$. For $x_{TF}(t)$ with a given peak time, we then minimized a mean squared error between the simulated $x_p(t)$ and the cuspidate profile in Fig 4B by optimization of parameters α's and λ's in Eq. (S88) and (S89).

S8A–S8L Fig shows that an inhibitor can generate a cuspidate profile of the target gene's protein (i.e., small RMSEs) if the inhibitor has a large phase difference (~8 to 12 hours) with the target gene's protein. In this case, the inhibitor's waveform can be either cuspidate (S8D Fig) or not (S8A, S8G, and S8J Fig). The non-cuspidate inhibitor waveforms are easily generated through common expression mechanisms, or through the superposition of a few waveforms (S8G and S8J Fig) when the inhibitor represents the combined activity of multiple inhibitors regulating the same gene [remind that Eq. (S88) and (S89) is not only valid for a single, but also for multiple transcription factors]. On the other hand, S8M and S8O Fig shows that if the inhibitor waveform is too sharp, it is difficult to make a cuspidate profile of the target gene's protein. In fact, we rarely observe such waveforms from the *Arabidopsis* clock components (S1–S5 Fig).

In the case of an activator, S8A–S8F and S8M–S8O Fig shows that the target gene's protein can have a cuspidate profile (i.e., small RMSEs) if the activator has a small phase difference ($\lesssim$ 4 hours) with the target gene's protein. In this case, the activator's waveform can be either cuspidate (S8E and S8N Fig) or not (S8B Fig), but should not have too long an acrophase duration (S8H and S8K Fig). It is also worthwhile to note that, as mentioned above, the transcription factor profile in S8N Fig is too sharp to be commonly observed from real clock components (S1–S5 Fig).

These results suggest that an inhibitor (activator) and its target have an approximately antiphase-like (inphase-like) relationship, giving rise to large (small) phase differences between them. It is possible that activating (inhibitory) regulation also induces larger (smaller) phase differences between the genes, but fails to generate cuspidate profiles in these cases (S9 Fig).

The above intriguing connection between a cuspidate waveform and inhibitory/activating regulation was observed in our simulation with the artificial protein expression profiles depicted in S8 Fig. Similar simulation results were obtained using real experimental profiles. S10 Fig shows the simulation results with LHY and PRR5 experimental profiles, which are both cuspidate in 12L:12D cycles (a 12L:12D condition was the only light condition with both available LHY and PRR5 experimental profiles). S10 Fig shows that the real experimental profiles support our claim for the relationship between cuspidate waveforms and inhibitory/activating interactions. Moreover, instead of Fig 4B, use of its slightly smoothened waveform (like 4-hour sampling intervals) did not change our results much.

Inhibition-induced large phase differences between genes correspond to the temporal coordination of multiple clock events with peaks that are temporally distant from each other. Conversely, activation-induced small phase differences between genes may only coordinate clock events that are temporally close. In other words, inhibitory interactions in the plant clock seem to benefit the global coordination of multiple phase-specific events over time. However, it should be stressed that inhibitory interactions do not necessarily result in cuspidate waveforms in all cases. Rather, obtaining such waveform profiles requires inhibitory interactions when involving genes with large phase differences in their peak expression. Employing the terms in propositional logic, the presence of both cuspidate waveforms and large phase differences is close to a sufficient condition to implicate inhibitory regulation as their cause, but is not the necessary condition.

Generally, it is known that dynamical systems with only activating interactions do not easily generate oscillations; inhibitory interactions are also necessary. Specifically, an odd number of inhibitory interactions need to be arranged along a feedback loop, if the loop is not too long. In addition to this basal level of inhibitory interactions required, an abundance of cuspidate-waveform genes in the plant circadian oscillator tips the balance in favor of a greater number of inhibitory interactions, resulting in their dominance, according to our hypothesis.

S4.2. Revisiting *PRR5* inhibition by LHY/CCA1

In Section S3.3, *PRR5* inhibition by LHY/CCA1 was found to be placed in opposition to the loop I’s overall cyclic direction, and thus antagonistic to the loop’s oscillatory capability. Despite this antagonistic effect, we suggest that the *PRR5* inhibition by LHY/CCA1 should be present for the

formation of the cuspidate PRR5 profiles (S1, S2, S4, and S5 Fig). In MF2015, LHY/CCA1 inhibits *PRR5* expression ~17 times more strongly than TOC1 [in Eq. (S8), $\theta^2_{44}/\theta^2_{45}$ = 16.9 from S2 Table].

The importance of the inhibition by LHY/CCA1 for the cuspidate waveform of PRR5 is proven by examining the PRR5 profile when reducing the inhibition strength. S11 Fig shows the results: we decreased the inhibition by 20% and 30% in MF2015 [i.e., multiplied $c^p_L(t)^2$ in Eq. (S8) by 0.8 and 0.7, respectively] while controlling for peak height and period of the PRR5 profile. To maintain the same peak height as the original, we increased the *PRR5* inhibition by TOC1 by 21% and 37%, respectively [i.e., multiplied $c^p_T(t)^2$ in Eq. (S8) by 1.21 and 1.37, respectively]. To maintain the same period as the original, we performed this simulation in 12L:12D cycles. The results show that a peak-to-trough change in PRR5 expression over time is reduced when decreasing the inhibition by LHY/CCA1 (S11 Fig). In other words, the waveform of PRR5 became less cuspidate, demonstrating the importance of the inhibition by LHY/CCA1 for the PRR5 waveform.

References

1. Ljung L. System Identification: Theory for the User, 2nd Edition. Englewood Cliff, NJ: Prentice Hall; 1999.
2. Foo M, Ooi SK, Weyer E. System identification and control of the Broken River. IEEE Trans Control Syst Technol. 2014; 22:618-634. doi: 10.1109/TCST.2013.2253103
3. Young P. Data-based mechanistic modelling of environmental, ecological, economic and engineering systems. Environ Model Softw. 1998; 13:105-122. doi: 10.1016/S1364-8152(98)00011-5
4. Dalchau N, Hubbard KE, Robertson FC, Hotta CT, Briggs HM, Stan G-B, et al. Correct biological timing in *Arabidopsis* requires multiple light-signaling pathways. Proc Natl Acad Sci USA. 2010; 107:13171-13176. doi: 10.1073/pnas.1001429107 PMID: 20615944
5. Herrero E, Kolmos E, Bujdoso N, Yuan Y, Wang M, Berns MC, et al. EARLY FLOWERING4 recruitment of EARLY FLOWERING3 in the nucleus sustains the *Arabidopsis* circadian clock. Plant Cell. 2012; 24:428-443. doi: 10.1105/tpc.111.093807 PMID: 22327739
6. Locke JCW, Millar AJ, Turner MS. Modelling genetic networks with noisy and varied experimental data: the circadian clock in *Arabidopsis thaliana*. J Theor Biol. 2005; 234:383-393. doi: 10.1016/j.jtbi.2004.11.038
7. Zeilinger MN, Farré EM, Taylor SR, Kay SA, Doyle FJ. A novel computational model of the circadian clock in *Arabidopsis* that incorporates PRR7 and PRR9. Mol Syst Biol. 2006; 2:58. doi: 10.1038/msb4100101 PMID: 17102803
8. Pokhilko A, Fernández AP, Edwards KD, Southern MM, Halliday KJ, Millar AJ. The clock gene circuit in *Arabidopsis* includes a repressilator with additional feedback loops. Mol Syst Biol. 2012; 8:574. doi: 10.1038/msb.2012.6 PMID: 22395476
9. Pokhilko A, Mas P, Millar A. Modelling the widespread effects of TOC1 signalling on the plant circadian clock and its outputs. BMC Syst Biol. 2013; 7:23. doi: 10.1186/1752-0509-7-23 PMID: 23506153
10. Nusinow DA, Helfer A, Hamilton EE, King JJ, Imaizumi T, Schultz TF, et al. The ELF4-ELF3-LUX complex links the circadian clock to diurnal control of hypocotyl growth. Nature. 2011; 475:398-402. doi: 10.1038/nature10182 PMID: 21753751

11. Chen H, Shen Y, Tang X, Yu L, Wang J, Guo L, et al. *Arabidopsis* CULLIN4 forms an E3 ubiquitin ligase with RBX1 and the CDD complex in mediating light control of development. Plant Cell. 2006; 18:1991-2004. doi: 10.1105/tpc.106.043224
12. Yu J-W, Rubio V, Lee N-Y, Bai S, Lee S-Y, Kim S-S, et al. COP1 and ELF3 control circadian function and photoperiodic flowering by regulating GI stability. Mol Cell. 2008; 32:617-630. doi: 10.1016/j.molcel.2008.09.026 PMID: 19061637
13. Matsushika A, Makino S, Kojima M, Mizuno T. Circadian waves of expression of the APRR1/TOC1 family of pseudo-response regulators in *Arabidopsis thaliana*: Insight into the plant circadian clock. Plant Cell Physiol. 2000; 41:1002-1012. doi: 10.1093/pcp/pcd043
14. Farre EM, Harmer SL, Harmon FG, Yanovsky MJ, Kay SA. Overlapping and distinct roles of *PRR7* and *PRR9* in the *Arabidopsis c*ircadian clock. Curr Biol. 2005; 15:47-54. doi: 10.1016/j.cub.2004.12.067
15. Somers DE, Kim W-Y, Geng R. The F-Box protein ZEITLUPE confers dosage-dependent control on the circadian clock, photomorphogenesis, and flowering time. Plant Cell. 2004; 16:769-782. doi: 10.1105/tpc.016808 PMID: 14973171
16. Foo M, Somers D, Kim P-J. System identification of the *Arabidopsis* plant circadian system. J Korean Phys Soc. 2015; 66:700-712. doi: 10.3938/jkps.66.700
17. Ljung L, Glad T. Modeling of Dynamic Systems. Englewood Cliff, NJ: Prentice Hall; 1994.
18. Heck HdA. Statistical theory of cooperative binding to proteins. Hill equation and the binding potential. J Am Chem Soc. 1971; 93:23-29. doi: 10.1021/ja00730a004
19. Nakamichi N, Kiba T, Henriques R, Mizuno T, Chua N-H, Sakakibara H. PSEUDO-RESPONSE REGULATORS 9, 7, and 5 are transcriptional repressors in the *Arabidopsis* circadian clock. Plant Cell. 2010; 22:594-605. doi: 10.1105/tpc.109.072892 PMID: 20233950
20. Gendron JM, Pruneda-Paz JL, Doherty CJ, Gross AM, Kang SE, Kay SA. *Arabidopsis* circadian clock protein, TOC1, is a DNA-binding transcription factor. Proc Natl Acad Sci USA. 2012; 109:3167-3172. doi: 10.1073/pnas.1200355109 PMID: 22315425
21. Levine E, Hwa T. Stochastic fluctuations in metabolic pathways. Proc Natl Acad Sci USA. 2007; 104:9224-9229. doi: 10.1073/pnas.0610987104 PMID: 17517669

22. Fogelmark K, Troein C. Rethinking transcriptional activation in the *Arabidopsis c*ircadian clock. PLoS Comput Biol. 2014; 10:e1003705. doi: 10.1371/journal.pcbi.1003705 PMID: 25033214
23. Hsu PY, Devisetty UK, Harmer SL. Accurate timekeeping is controlled by a cycling activator in *Arabidopsis*. Elife. 2013; 2:e00473. doi: 10.7554/eLife.00473 PMID: 23638299
24. Kim W-Y, Geng R, Somers DE. Circadian phase-specific degradation of the F-box protein ZTL is mediated by the proteasome. Proc Natl Acad Sci USA. 2003; 100:4933-4938. doi: 10.1073/pnas.0736949100 PMID: 12665620
25. Reed R. Pruning algorithm-a survey. IEEE Trans Neural Netw. 1993; 4:740-747. doi: 10.1109/72.248452
26. Engelbrecht AP. A new pruning heuristic based on variance analysis of sensitivity information. IEEE Trans Neural Netw. 2001; 12:1386-1399. doi: 10.1109/72.963775
27. Elowitz MB, Leibler S. A synthetic oscillatory network of transcriptional regulators. Nature. 2000; 403:335-338. doi: 10.1038/35002125
28. Mockler TC, Michael TP, Priest HD, Shen R, Sullivan CM, Givan SA, et al. The diurnal project: diurnal and circadian expression profiling, model-based pattern matching, and promoter analysis. Cold Spring Harb Symp Quant Biol. 2007; 72:353-363. doi: 10.1101/sqb.2007.72.006
29. Baudry A, Ito S, Song YH, Strait AA, Kiba T, Lu S, et al. F-box proteins FKF1 and LKP2 act in concert with ZEITLUPE to control *Arabidopsis* clock progression. Plant Cell. 2010; 22:606-622. doi: 10.1105/tpc.109.072843
30. Kim J-Y, Song H-R, Taylor BL, Carré IA. Light-regulated translation mediates gated induction of the *Arabidopsis* clock protein LHY. EMBO J. 2003; 22:935-944. doi: 10.1093/emboj/cdg075 PMID: 12574129
31. Nakamichi N, Matsushika A, Yamashino T, Mizuno T. Cell autonomous circadian waves of the APRR1/TOC1 quintet in an established cell line of *Arabidopsis thaliana*. Plant Cell Physiol. 2003; 44:360-365. doi: 10.1093/pcp/pcg039
32. Farre EM, Kay SA. PRR7 protein levels are regulated by light and the circadian clock in *Arabidopsis*. Plant J. 2007; 52:548-560. doi: 10.1111/j.1365-313X.2007.03258.x
33. Kiba T, Henriques R, Sakakibara H, Chua NH. Targeted degradation of PSEUDO-RESPONSE REGULATOR5 by an SCFZTL complex regulates clock function and

photomorphogenesis in *Arabidopsis thaliana*. Plant Cell. 2007; 19:2516-2530. doi: 10.1105/tpc.107.053033 PMID: 17693530

34. Onai K, Ishiura M. PHYTOCLOCK 1 encoding a novel GARP protein essential for the *Arabidopsis* circadian clock. Genes Cells. 2005; 10:963-972. doi: 10.1111/j.1365-2443.2005.00892.x
35. Edwards KD, Akman OE, Knox K, Lumsden PJ, Thomson AW, Brown PE, et al. Quantitative analysis of regulatory flexibility under changing environmental conditions. Mol Syst Biol. 2010; 6:424. doi: 10.1038/msb.2010.81 PMID: 21045818
36. Kim W-Y, Fujiwara S, Suh S-S, Kim J, Kim Y, Han L, et al. ZEITLUPE is a circadian photoreceptor stabilized by GIGANTEA in blue light. Nature. 2007; 449:356-360. doi: 10.1038/nature06132
37. Farinas B, Mas P. Functional implication of the MYB transcription factor *RVE8/LCL5* in the circadian control of histone acetylation. Plant J. 2011; 66:318-329. doi: 10.1111/j.1365-313X.2011.04484.x
38. Liu XL, Covington MF, Fankhauser C, Chory J, Wagner DR. *ELF3* encodes a circadian clock–regulated nuclear protein that functions in an *Arabidopsis PHYB* signal transduction pathway. Plant Cell. 2001; 13:1293-1304. doi: 10.1105/TPC.000475
39. David KM, Armbruster U, Tama N, Putterill J. *Arabidopsis* GIGANTEA protein is post-transcriptionally regulated by light and dark. FEBS Lett. 2006; 580:1193-1197. doi: 10.1016/j.febslet.2006.01.016
40. Mizoguchi T, Wheatley K, Hanzawa Y, Wright L, Mizoguchi M, Song H-R, et al. *LHY* and *CCA1* are partially redundant genes required to maintain circadian rhythms in *Arabidopsis*. Dev Cell. 2002; 2:629-641. doi: 10.1016/S1534-5807(02)00170-3
41. Strayer C, Oyama T, Schultz TF, Raman R, Somers DE, Más P, et al. Cloning of the *Arabidopsis c*lock cene *TOC1*, an autoregulatory response regulator homolog. Science. 2000; 289:768-771. doi: 10.1126/science.289.5480.768
42. Somers DE, Schultz TF, Milnamow M, Kay SA. *ZEITLUPE* encodes a novel clock-associated PAS protein from *Arabidopsis*. Cell. 2000; 101:319-329. doi: 10.1016/S0092-8674(00)80841-7

43. Gould PD, Locke JCW, Larue C, Southern MM, Davis SJ, Hanano S, et al. The molecular basis of temperature compensation in the *Arabidopsis* circadian clock. Plant Cell. 2006; 18:1177-1187. doi: 10.1105/tpc.105.039990 PMID: 16617099
44. McWatters HG, Kolmos E, Hall A, Doyle MR, Amasino RM, Gyula P, et al. *ELF4* is required for oscillatory properties of the circadian clock. Plant Physiol. 2007; 144:391-401. doi: 10.1104/pp.107.096206 PMID: 17384164
45. McWatters HG, Bastow RM, Hall A, Millar AJ. The *ELF3 zeitnehmer* regulates light signalling to the circadian clock. Nature. 2000; 408:716-720. doi: 10.1038/35047079
46. Martin-Tryon EL, Kreps JA, Harmer SL. *GIGANTEA* acts in blue light signaling and has biochemically separable roles in circadian clock and flowering time regulation. Plant Physiol. 2007; 143:473-486. doi: 10.1104/pp.106.088757 PMID: 17098855
47. Covington MF, Panda S, Liu XL, Strayer CA, Wagner DR, Kay SA. ELF3 modulates resetting of the circadian clock in *Arabidopsis*. Plant Cell. 2001; 13:1305-1316. doi: 10.1105/TPC.000561
48. Doyle MR, Davis SJ, Bastow RM, McWatters HG, Kozma-Bognar L, Nagy F, et al. The *ELF4* gene controls circadian rhythms and flowering time in *Arabidopsis thaliana*. Nature. 2002; 419:74-77. doi: 10.1038/nature00954
49. Kevei É, Gyula P, Hall A, Kozma-Bognár L, Kim W-Y, Eriksson ME, et al. Forward genetic analysis of the circadian clock separates the multiple functions of *ZEITLUPE*. Plant Physiol. 2006; 140:933-945. doi: 10.1104/pp.105.074864 PMID: 16428597
