# Supplementary figures and images for: Kernel Architecture of the Genetic Circuitry of the Arabidopsis Circadian System
Source: PLoS Comput Biol. 2016 Feb 1;12(2):e1004748. doi: 10.1371/journal.pcbi.1004748 (PMC4734688; doi:10.1371/journal.pcbi.1004748)

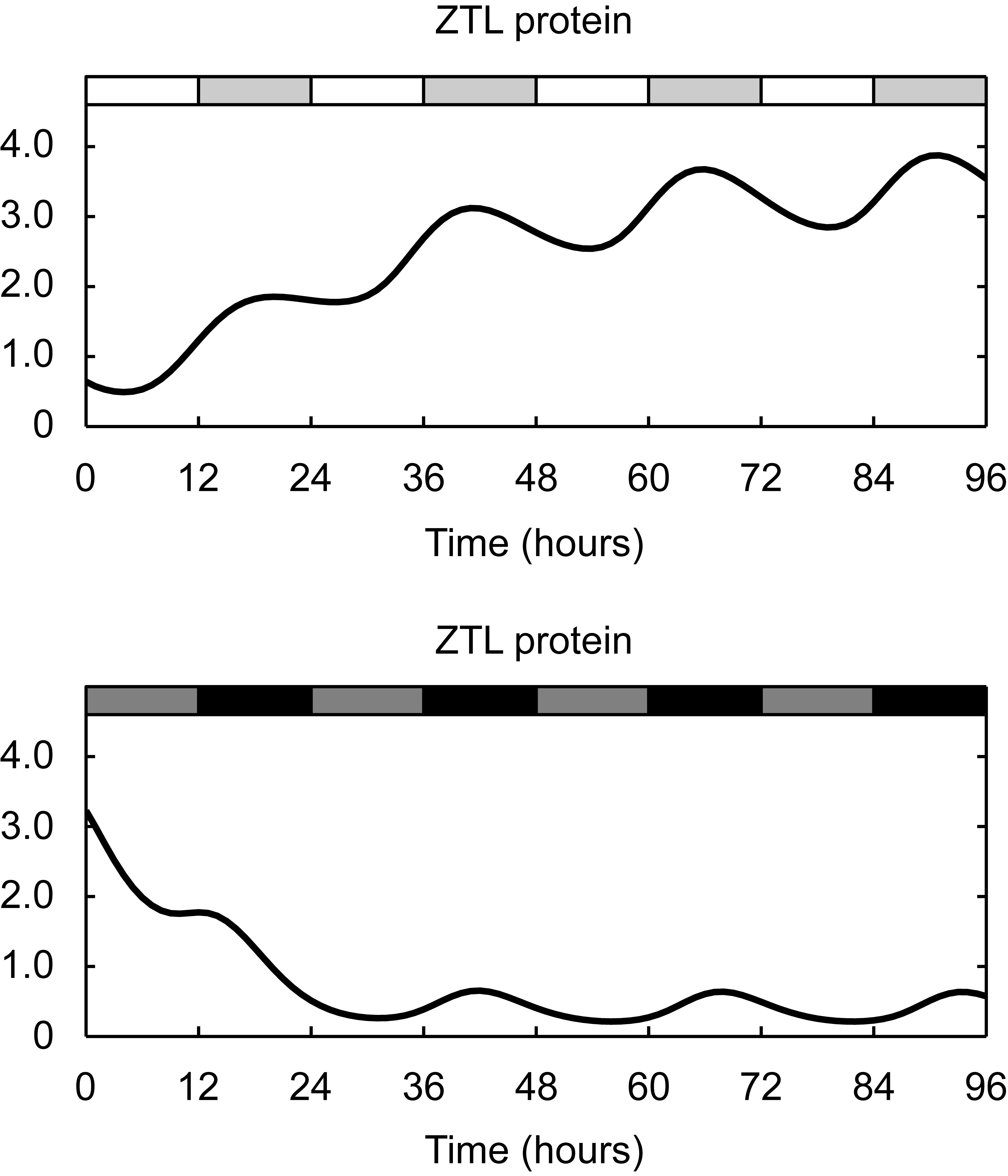

Supplement: S6 Fig — Initially entrained over four 12L:12D cycles and released into either LL or DD. (TIF) [file pcbi.1004748.s007.tif]

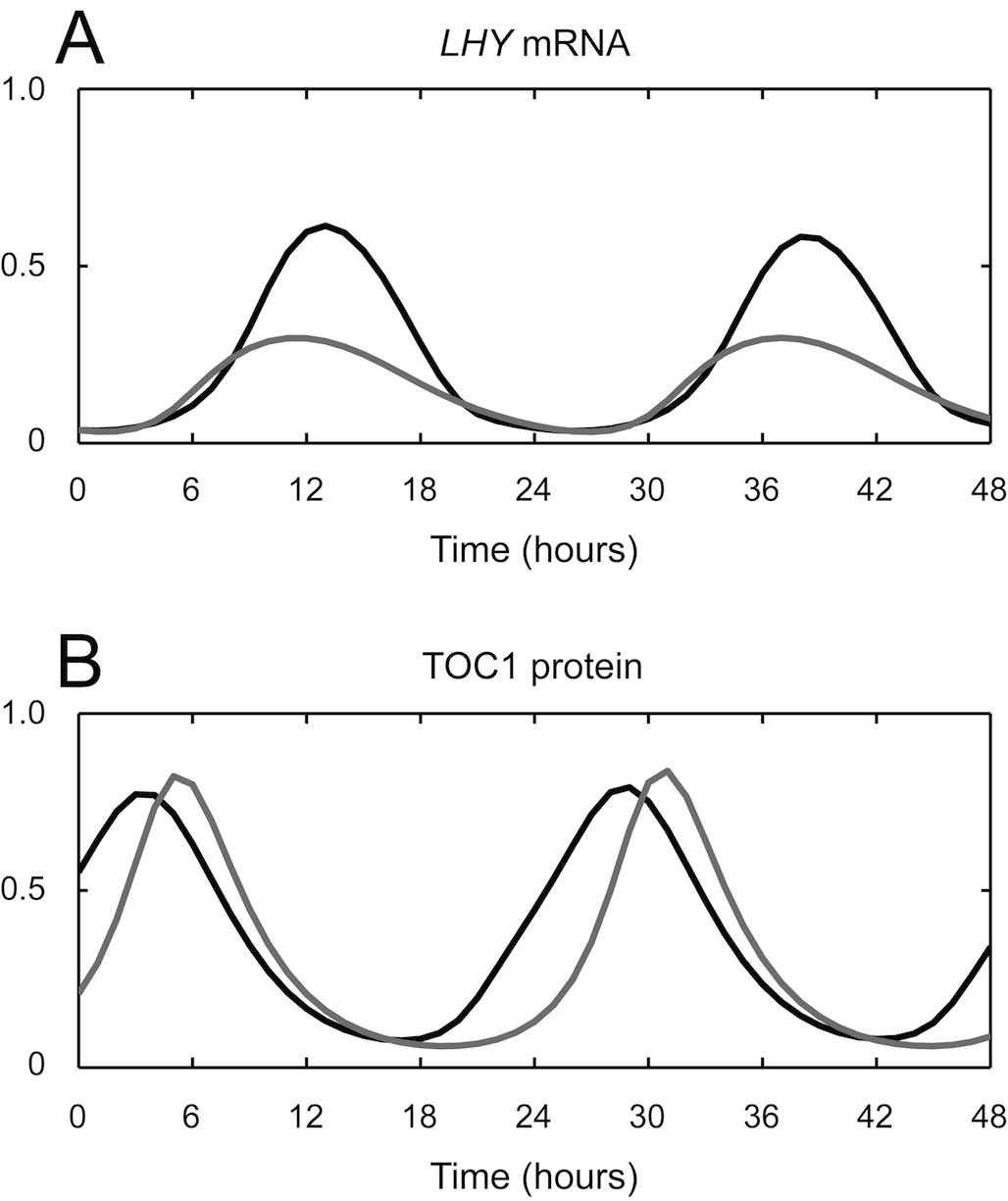

Supplement: S7 Fig — The latter was accompanied by parameter re-optimization. Initially entrained over four 12L:12D cycles and released into LL. (TIF) [file pcbi.1004748.s008.tif]

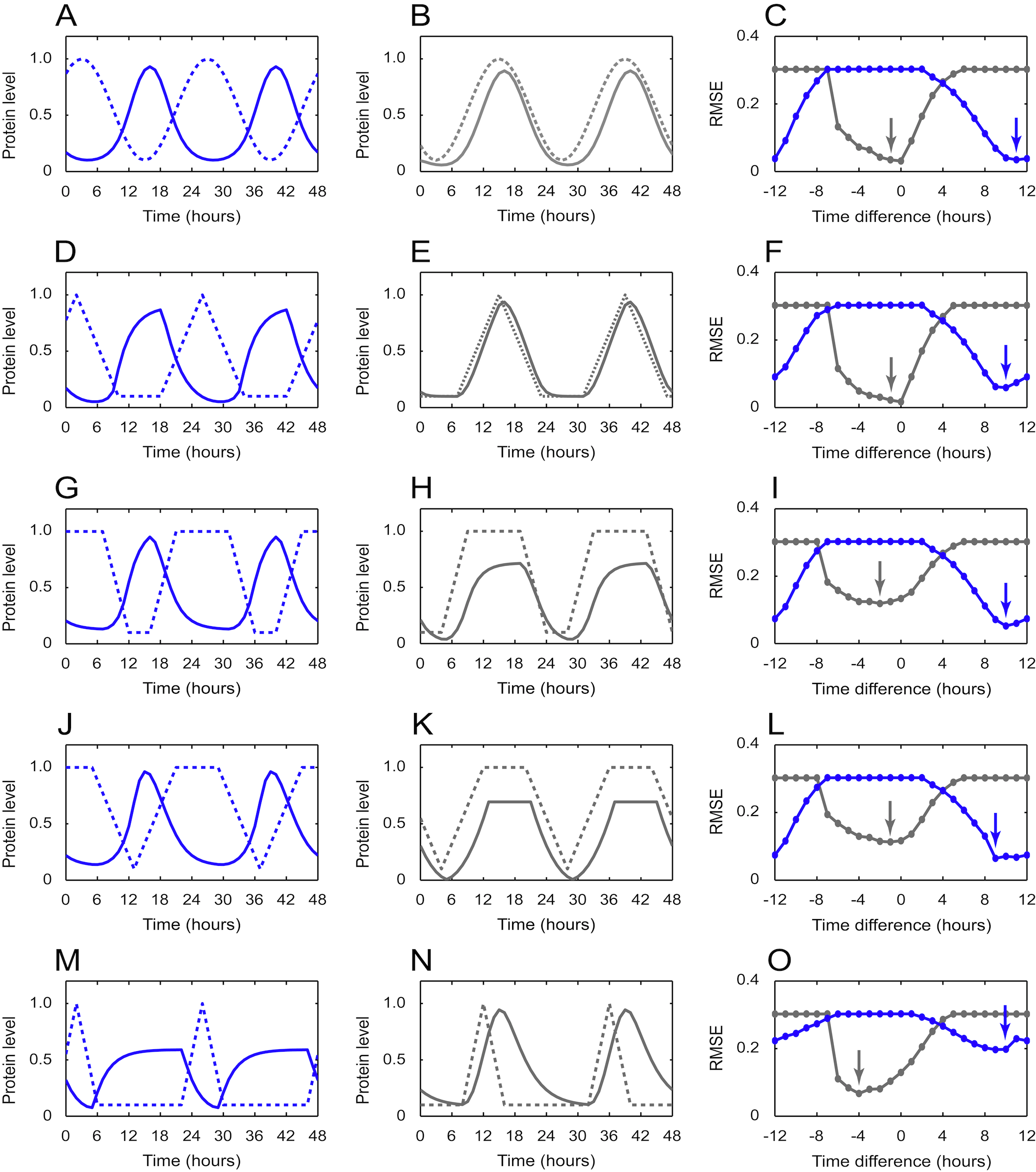

Supplement: S8 Fig — Simulation details are presented in S1 Text. The left and center panels show diverse profiles of an inhibitor (dotted in the left panel) or activator (dotted in the center panel) that regulates the target gene to produce its proteins (solid). Given the inhibitor or activator levels, the target gene transcription, translation, and product degradation were simulated with the parameters that best fit the desired, cuspidate profile in Fig 4B. For a given phase difference between a transcription factor (inhibitor or activator) and its target gene’s protein, the right panels plot the inevitable deviation of the target gene’s protein profile from the cuspidate profile. The horizontal axis represents the phase difference between each transcription factor’s profile and the target gene’s desired protein profile in Fig 4B; a sign is negative if the former profile has a more advanced phase than the latter, and otherwise, it is positive. The vertical axis represents the root mean square error (RMSE) between the target gene’s actual and desired protein profiles. The target gene expression was simulated with the parameters that best fit the cuspidate profile in Fig 4B: the smaller the RMSE, the more cuspidate profile the target gene has. An inhibitor case is shown in blue, and an activator case is shown in gray. Arrows in blue and gray correspond to the conditions for the left and center panels, respectively. (TIF) [file pcbi.1004748.s009.tif]

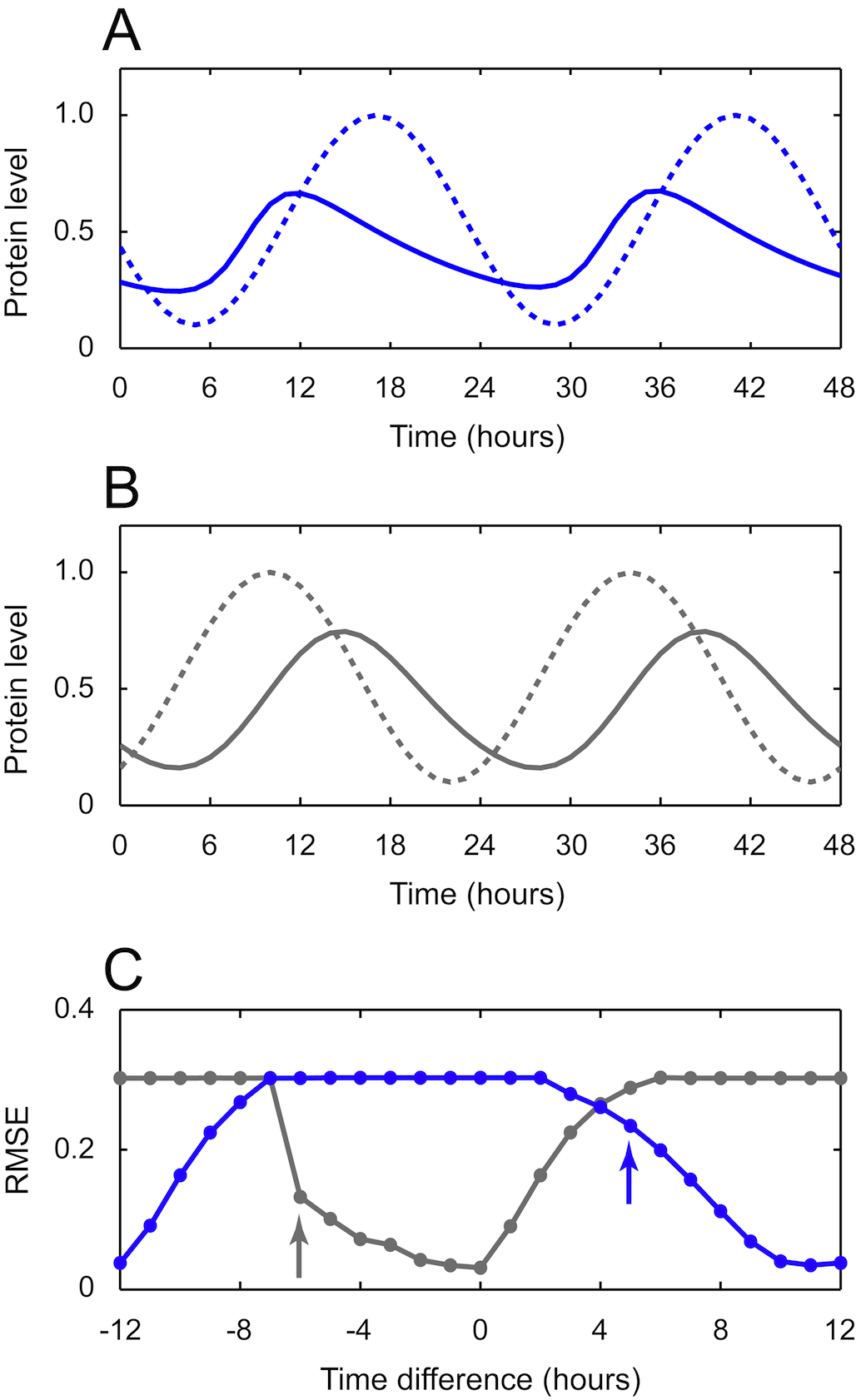

Supplement: S9 Fig — The resulting RMSEs are large as arrowed in (C). Plotted according to S8A–S8C Fig. (TIF) [file pcbi.1004748.s010.tif]

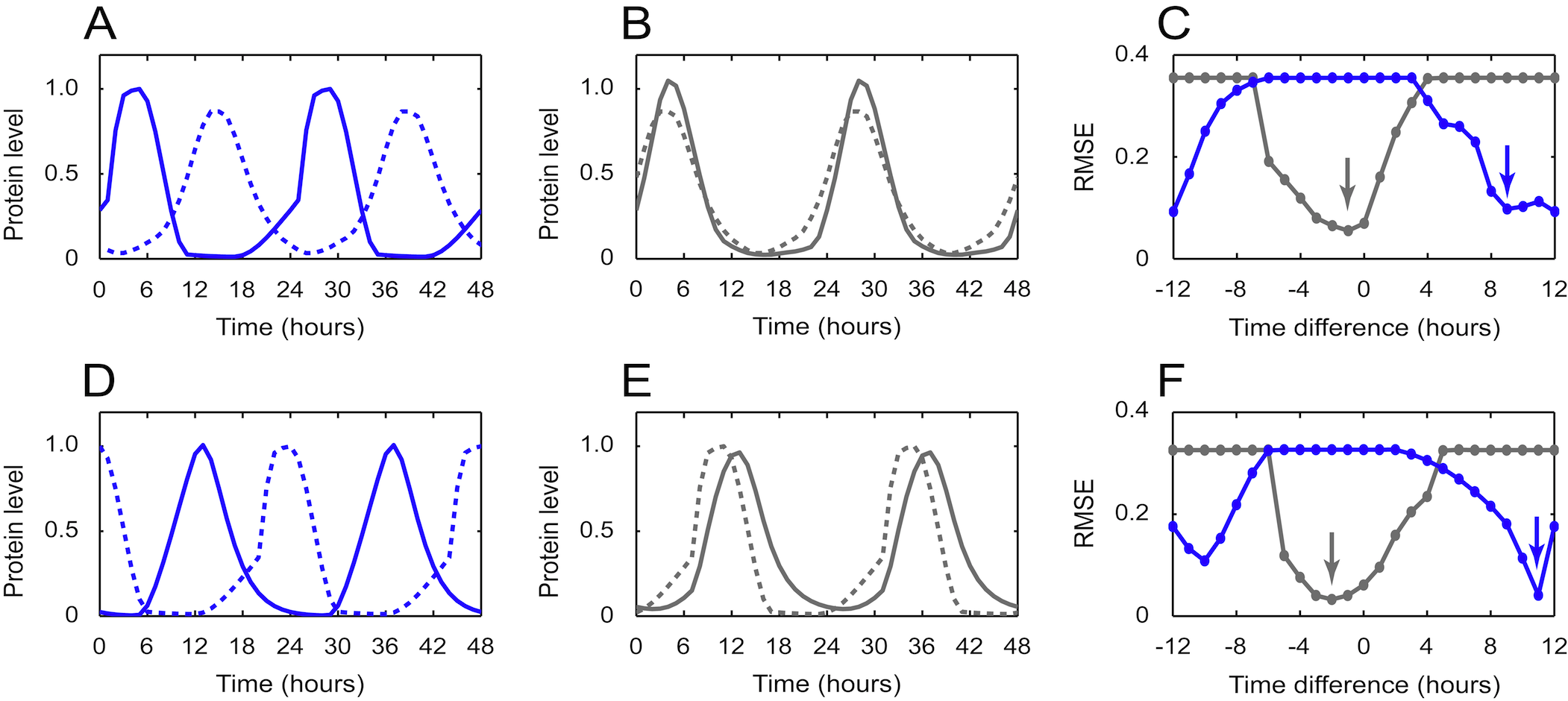

Supplement: S10 Fig — (A–C) The PRR5 waveform was used for the transcription factor’s profile, and the LHY waveform was used for the desired cuspidate profile of the target gene’s protein. (D–F) The LHY waveform was used for the transcription factor’s profile, and the PRR5 waveform was used for the desired cuspidate profile of the target gene’s protein. For (A–F), we adopted the LHY and PRR5 experimental profiles in 12L:12D cycles (a 12L:12D condition was the only light condition with both available LHY and PRR5 experimental profiles). (TIF) [file pcbi.1004748.s011.tif]

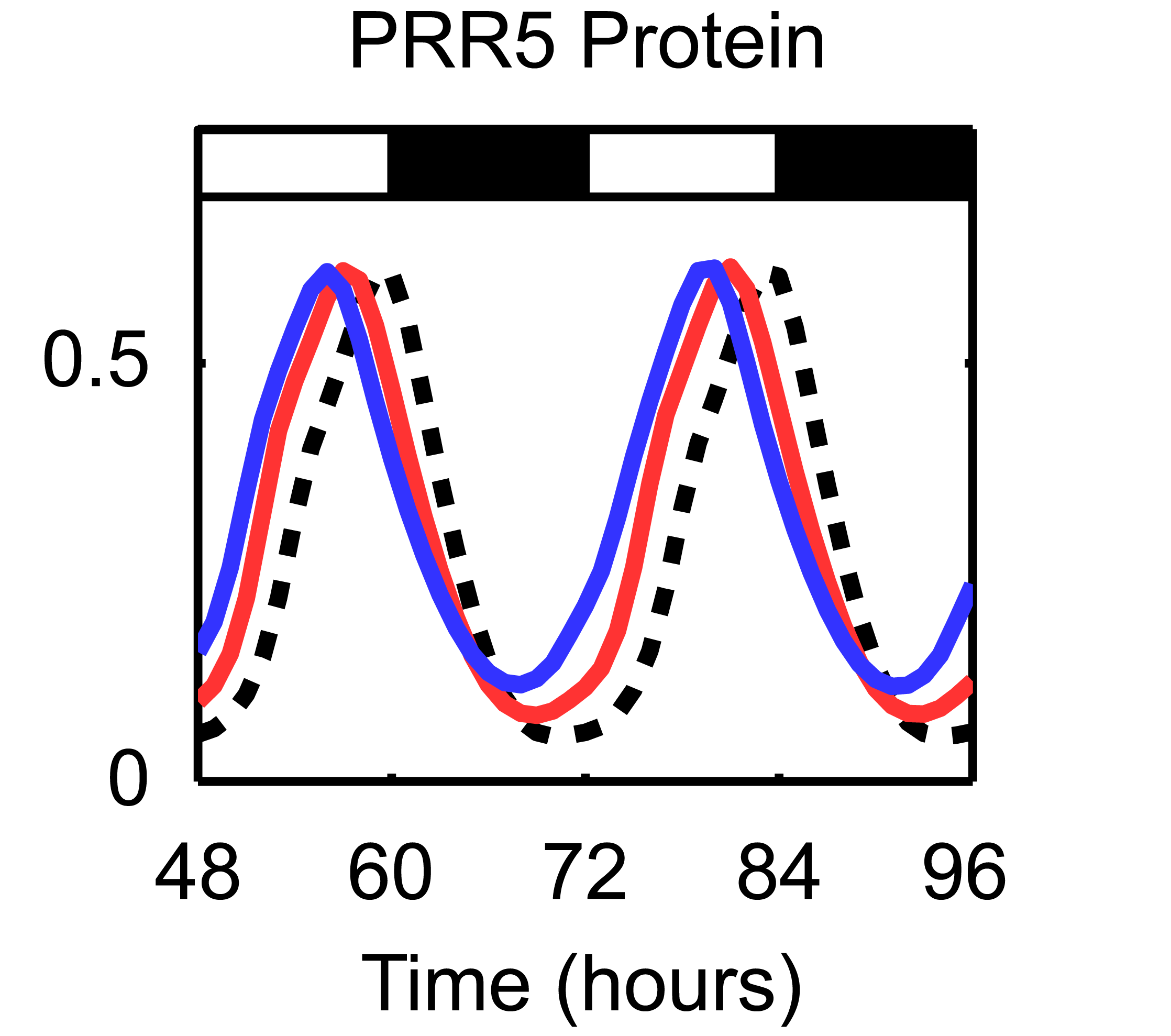

Supplement: S11 Fig — In parallel, the PRR5 inhibition by TOC1 was increased to control for the peak height of PRR5 levels. Simulation details are presented in S1 Text. (TIF) [file pcbi.1004748.s012.tif]
