# Supplementary material for: Kernel Architecture of the Genetic Circuitry of the Arabidopsis Circadian System
Source: PLoS Comput Biol. 2016 Feb 1;12(2):e1004748. doi: 10.1371/journal.pcbi.1004748 (PMC4734688; doi:10.1371/journal.pcbi.1004748)
Supplement: S1 Table — (PDF) [file pcbi.1004748.s013.pdf]

S1 Table. Sources of mRNA and protein expression data. References are available in S1 Text.

mRNA/protein	Light condition	Source
LHY mRNA	12L:12D cycle	Mockler et al. [28]
	Short day	Mockler et al. [28]
	Long day	Mockler et al. [28]
	LL	Baudry et al. [29]
	DD	Mockler et al. [28]
LHY protein	12L:12D cycle	Kim et al. [30]
	Short day	Mockler et al. [28]
	Long day	Mockler et al. [28]
PRR9 mRNA	12L:12D cycle	Nakamichi et al. [31]
	Short day	Mockler et al. [28]
	Long day	Mockler et al. [28]
	LL	Baudry et al. [29]
	DD	Nakamichi et al. [31]
PRR9 protein	12L:12D cycle	Nakamichi et al. [19]
PRR7 mRNA	12L:12D cycle	Mockler et al. [28]
	Short day	Mockler et al. [28]
	Long day	Mockler et al. [28]
	LL	Farre & Kay [32]
	DD	Nakamichi et al. [31]
PRR7 protein	12L:12D cycle	Nakamichi et al. [19]
	LL	Farre & Kay [32]
PRR5 mRNA	12L:12D cycle	Mockler et al. [28]
	Short day	Mockler et al. [28]
	Long day	Mockler et al. [28]
	LL	Hsu et al. [23]
	DD	Mockler et al. [28]

S1 Table. (Continued)

mRNA/protein	Light condition	Source
PRR5 protein	12L:12D cycle	Nakamichi et al. [19]
	Short day	Kiba et al. [33]
	Long day	Kiba et al. [33]
	LL	Baudry et al. [29]
	DD	Kiba et al. [33]
TOC1 mRNA	12L:12D cycle	Mockler et al. [28]
	Short day	Mockler et al. [28]
	Long day	Mockler et al. [28]
	LL	Onai & Ishiura [34]
	DD	Edwards et al. [35]
TOC1 protein	12L:12D cycle	Kim et al. [36]
	LL	Baudry et al. [29]
RVE8 mRNA	12L:12D cycle	Hsu et al. [23]
	Short day	Mockler et al. [28]
	Long day	Mockler et al. [28]
	LL	Farinas & Mas [37]
RVE8 protein	12L:12D cycle	Hsu et al. [23]
ELF3 mRNA	12L:12D cycle	Mockler et al. [28]
	Short day	Mockler et al. [28]
	Long day	Mockler et al. [28]
	LL	Liu et al. [38]
ELF3 protein	12L:12D cycle	Nusinow et al. [10]
	Short day	Yu et al. [12]
	Long day	Yu et al. [12]
	LL	Liu et al. [38]

S1 Table. (Continued)

mRNA/protein	Light condition	Source
ELF4 mRNA	12L:12D cycle	Mockler et al. [28]
	Short day	Mockler et al. [28]
	Long day	Mockler et al. [28]
	LL	Onai & Ishiura [34]
	DD	Mockler et al. [28]
ELF4 protein	12L:12D cycle	Nusinow et al. [10]
	LL	Nusinow et al. [10]
LUX mRNA	12L:12D cycle	Mockler et al. [28]
	Short day	Mockler et al. [28]
	Long day	Mockler et al. [28]
	LL	Onai & Ishiura [34]
	DD	Mockler et al. [28]
LUX protein	12L:12D cycle	Nusinow et al. [10]
	LL	Nusinow et al. [10]
GI mRNA	12L:12D cycle	Mockler et al. [28]
	Short day	Mockler et al. [28]
	Long day	Mockler et al. [28]
	LL	Onai & Ishiura [34]
	DD	Edwards et al. [35]
GI protein	12L:12D cycle	Kim et al. [36]
	Short day	David et al. [39]
	Long day	David et al. [39]
ZTL protein	12L:12D day	Kim et al. [36]
