# Supplementary material for: Kernel Architecture of the Genetic Circuitry of the Arabidopsis Circadian System
Source: PLoS Comput Biol. 2016 Feb 1;12(2):e1004748. doi: 10.1371/journal.pcbi.1004748 (PMC4734688; doi:10.1371/journal.pcbi.1004748)
Supplement: S2 Table — (PDF) [file pcbi.1004748.s014.pdf]

**S2 Table. Estimated parameters of the MF2015 model.** Refer to Eq. (S1)–(S24) in S1 Text.

| mRNA/Protein/Scaling factor | Parameter value |
|---|---|
| Protein P | $\theta_1$ = -0.43301, $\theta_2$ = -0.4949, $\theta_3$ = 0.4358 |
| *LHY* mRNA | $\theta_4$ = 0.24679, $\theta_5$ = 1.8815, $\theta_6$ = 0.79145 |
| | $\theta_7$ = 0.33897, $\theta_8$ = -2.7692 |
| *LHY* mRNA scaling factor | $\theta_9$ = 0.62302 (short day), $\theta_{10}$ = 0.71035 (long day), $\theta_{11}$ = 0.60589 (LL), $\theta_{12}$ = 0.86705 (DD) |
| LHY protein | $\theta_{13}$ = 0.8825, $\theta_{14}$ = -0.69509 |
| *PRR9* mRNA | $\theta_{15}$ = 0.72904, $\theta_{16}$ = 0.037655, $\theta_{17}$ = 0.37213 |
| | $\theta_{18}$ = 21.1469, $\theta_{19}$ = 7.8575, $\theta_{20}$ = 0.91021 |
| | $\theta_{21}$ = 2.0415, $\theta_{22}$ = -0.39927 |
| *PRR9* mRNA scaling factor | $\theta_{23}$ = 0.72436 (short day), $\theta_{24}$ = 0.71495 (long day), $\theta_{25}$ = 0.2751 (LL), $\theta_{26}$ = 0.094967 (DD) |
| PRR9 protein | $\theta_{27}$ = 0.33303, $\theta_{28}$ = -0.1134 |
| PRR7 mRNA | $\theta_{29}$ = 0.39385, $\theta_{30}$ = 0.030188, $\theta_{31}$ = 6.5356 |
| | $\theta_{32}$ = 0.00059033, $\theta_{33}$ = 6.2504e-05, |
| | $\theta_{34}$ = 4.0393, $\theta_{35}$ = -0.25102 |
| *PRR7* mRNA scaling factor | $\theta_{36}$ = 0.87088 (short day), $\theta_{37}$ = 0.85927 (long day), $\theta_{38}$ = 0.99951 (LL), $\theta_{39}$ = 0.6672 (DD) |
| PRR7 protein | $\theta_{40}$ = 0.24208, $\theta_{41}$ = -0.17782 |
| PRR7 protein scaling factor | $\theta_{42}$ = 0.83682 (LL) |
| *PRR5* mRNA | $\theta_{43}$ = 0.029094, $\theta_{44}$ = 0.11658, $\theta_{45}$ = 0.028392 |
| | $\theta_{46}$ = 0.70684, $\theta_{47}$ = 46.5777, $\theta_{48}$ = -41.5486 |
| *PRR5* mRNA scaling factor | $\theta_{49}$ = 0.53738 (short day), $\theta_{50}$ = 0.54906 (long day), $\theta_{51}$ = 0.45036 (LL), $\theta_{52}$ = 0.29048 (DD) |
| PRR5 protein | $\theta_{53}$ = 3.6953, $\theta_{54}$ = -0.00016248, $\theta_{55}$ = -2.6766 |
| PRR5 protein scaling factor | $\theta_{56}$ = 0.65048 (short day), $\theta_{57}$ = 0.47886 (long day), $\theta_{58}$ = 0.69446 (LL), $\theta_{59}$ = 0.56939 (DD) |

**S2 Table. (Continued)**

| mRNA/Protein/Scaling factor | Parameter value |
|---|---|
| *TOC1* mRNA | $\theta_{60}$ = 0.014431, $\theta_{61}$ = 0.038796, $\theta_{62}$ = 0.01094 $\theta_{63}$ = 1.323e-05, $\theta_{64}$ = 1.7345, $\theta_{65}$ = -12.7287 |
| *TOC1* mRNA scaling factor | $\theta_{66}$ = 0.85213 (short day), $\theta_{67}$ = 0.82656 (long day), $\theta_{68}$ = 0.65883 (LL), $\theta_{69}$ = 0.55127 (DD) |
| TOC1 protein | $\theta_{70}$ = 0.37371, $\theta_{71}$ = -0.000439, $\theta_{72}$ = -0.30649 |
| TOC1 protein scaling factor | $\theta_{73}$ = 0.70941 (LL) |
| EC complex | $\theta_{74}$ = 0.72174, $\theta_{75}$ = -0.47537 |
| *RVE8* mRNA | $\theta_{76}$ = 2.4874, $\theta_{77}$ = 1.2071, $\theta_{78}$ = 0.40419, $\theta_{79}$ = 1.3929, $\theta_{80}$ = 9.5812, $\theta_{81}$ = -1.48 |
| RVE8 mRNA scaling factor | $\theta_{82}$ = 0.59742 (short day), $\theta_{83}$ = 0.73739 (long day), $\theta_{84}$ = 0.68799 (LL) |
| RVE8 protein | $\theta_{85}$ = 0.23818, $\theta_{86}$ = -0.096012 |
| *ELF3* mRNA | $\theta_{87}$ = 0.023906, $\theta_{88}$ = 0.27988, $\theta_{89}$ = -0.32952 |
| *ELF3* mRNA scaling factor | $\theta_{90}$ = 0.87933 (short day), $\theta_{91}$ = 0.85884 (long day), $\theta_{92}$ = 0.8272 (LL) |
| ELF3 protein | $\theta_{93}$ = 15.9119, $\theta_{94}$ = -3.4184, $\theta_{95}$ = -17.2049 |
| ELF3 protein scaling factor | $\theta_{96}$ = 0.72758 (short day), $\theta_{97}$ = 0.85035 (long day), $\theta_{98}$ = 0.72698 (LL) |
| *ELF4* mRNA | $\theta_{99}$ = 0.014613, $\theta_{100}$ = 0.034975, $\theta_{101}$ = 0.056005, $\theta_{102}$ = 5.3297, $\theta_{103}$ = 15.9488, $\theta_{104}$ = -12.0676 |
| *ELF4* mRNA scaling factor | $\theta_{105}$ = 0.56392 (short day), $\theta_{106}$ = 0.67273 (long day), $\theta_{107}$ = 0.43802 (LL), $\theta_{108}$ = 0.39693 (DD) |
| ELF4 protein | $\theta_{109}$ = 0.56848, $\theta_{110}$ = -0.32213 |
| ELF4 protein scaling factor | $\theta_{111}$ = 0.8267 (LL) |

**S2 Table. (Continued)**

| mRNA/Protein/Scaling factor | Parameter value |
|---|---|
| *LUX* mRNA | $\theta_{112}$ = 0.030182, $\theta_{113}$ = 0.021833, $\theta_{114}$ = 0.037709, $\theta_{115}$ = 0.00060482, $\theta_{116}$ = 2.9989, $\theta_{117}$ = -28.1989 |
| *LUX* mRNA scaling factor | $\theta_{118}$ = 0.75222 (short day), $\theta_{119}$ = 0.6454 (long day), $\theta_{120}$ = 0.58704 (LL), $\theta_{121}$ = 0.51633 (DD) |
| LUX protein | $\theta_{122}$ = 2.6341, $\theta_{123}$ = -1.6287 |
| LUX protein scaling factor | $\theta_{124}$ = 1.1217 (LL) |
| *GI* mRNA | $\theta_{125}$ = 0.00088598, $\theta_{126}$ = 0.000262, $\theta_{127}$ = 0.10428, $\theta_{128}$ = 0.1132, $\theta_{129}$ = 0.45607, $\theta_{130}$ = -0.5337 |
| *GI* mRNA scaling factor | $\theta_{131}$ = 0.57545 (short day), $\theta_{132}$ = 0.62092 (long day), $\theta_{133}$ = 0.63741 (LL), $\theta_{134}$ = 0.49762 (DD) |
| GI protein | $\theta_{135}$ = 0.28193, $\theta_{136}$ = -0.38633, $\theta_{137}$ = -0.015299 |
| GI protein scaling factor | $\theta_{138}$ = 0.6958 (short day), $\theta_{139}$ = 1.1391 (long day) |
| COP1 protein | $\theta_{140}$ = -0.80938, $\theta_{141}$ = -0.8102, $\theta_{142}$ = 0.91424 |
| ZTL protein | $\theta_{143}$ = 0.013986, $\theta_{144}$ = 1.275, $\theta_{145}$ = 1.5983 $\theta_{146}$ = 1.9346, $\theta_{147}$ = -0.37054, $\theta_{148}$ = 0.023356 |
