# Supplementary material for: Kernel Architecture of the Genetic Circuitry of the Arabidopsis Circadian System
Source: PLoS Comput Biol. 2016 Feb 1;12(2):e1004748. doi: 10.1371/journal.pcbi.1004748 (PMC4734688; doi:10.1371/journal.pcbi.1004748)
Supplement: S3 Table — (PDF) [file pcbi.1004748.s015.pdf]

S3 Table. Free running periods of experimental and simulated mutants under constant conditions. Arr., arrhythmic; -OX, overexpression. References are available in S1 Text.

Mutant	Light condition	Period (Exp.)	Source	Period (Simulated)
Δlhy/cca1	LL	17.6	Mizoguhi et al. [40]	18.9
Δtoc1	LL	21.0	Strayer et al. [41]	23.3
Δprr7	LL	25.9	Farre et al. [14]	30.1
Δprr9	LL	25.3	Farre et al. [14]	26.9
Δprr9/prr7	LL	29.6	Farre et al. [14]	32.0
Δztl	LL	27.0	Somers et al. [42]	26.9
Δgi	LL	23.4	Gould et al. [43]	26.9
Δlux	LL	Arr.	Onai & Ishiura [34]	Arr.
Δelf4	LL	Arr.	McWatters et al. [44]	Arr.
Δelf3	LL	Arr.	McWatters et al. [45]	Arr.
Δrve8	LL	25.7	Farinas & Mas [37]	26.9
PRR5-OX	LL	22.7	Baudry et al. [29]	22.5
ELF4-OX	LL	28.8	Herrero et al. [5]	25.3
ELF3-OX	LL	26.8	Herrero et al. [5]	27.7
RVE8-OX	LL	22.2	Farinas & Mas [37]	24.5
Δgi	DD	Arr.	Martin-Tyron et al. [46]	26.9
Δelf3	DD	25.4	Covington et al. [47]	Arr.
Δelf4	DD	Arr.	Doyle et al. [48]	Arr.
Δztl	DD	33.6	Kevei et al. [49]	26.9
Δprr7	DD	25.8	Farre et al. [14]	34.1
ELF3-OX	DD	29.5	Herrero et al. [5]	27.1
