# Supplementary material for: Kernel Architecture of the Genetic Circuitry of the Arabidopsis Circadian System
Source: PLoS Comput Biol. 2016 Feb 1;12(2):e1004748. doi: 10.1371/journal.pcbi.1004748 (PMC4734688; doi:10.1371/journal.pcbi.1004748)
Supplement: S4 Table — (PDF) [file pcbi.1004748.s016.pdf]

S4 Table. Estimated parameters of the kernel structure. Refer to Eq. (S27)–(S50) in S1 Text.

mRNA/Protein	Parameter value
Protein P	ϕ_1 = -0.43301, ϕ_2 = -0.4949, ϕ_3 = 0.4358
LHY mRNA	ϕ_4 = 1.9601, ϕ_5 = 0.7412, ϕ_6 = 0.3821,
	ϕ_7 = 0.52644, ϕ_8 = 1.3764, ϕ_9 = 11.6684,
	ϕ_{10} = -2.7804
LHY protein	ϕ_{11} = 0.83604, ϕ_{12} = -0.64719
PRR9 mRNA	ϕ_{13} = 0.73369, ϕ_{14} = 0.00006, ϕ_{15} = 0.75976
	ϕ_{16} = 1.3352, ϕ_{17} = -0.59706
PRR9 protein	ϕ_{18} = 0.50077, ϕ_{19} = -0.22337
PRR7 mRNA	ϕ_{20} = 0.0099478, ϕ_{21} = 0.034706, ϕ_{22} = 0.87726
	ϕ_{23} = -0.23934
PRR7 protein	ϕ_{24} = 0.23744, ϕ_{25} = -0.18571
PRR5 mRNA	ϕ_{26} = 0.026728, ϕ_{27} = 0.13201, ϕ_{28} = 0.031588
	ϕ_{29} = -40.9795
PRR5 protein	ϕ_{30} = 4.0753, ϕ_{31} = -2.5872
TOC1 mRNA	ϕ_{32} = 0.01501, ϕ_{33} = 0.036926, ϕ_{34} = 0.011307
	ϕ_{35} = -12.2192
TOC1 protein	ϕ_{36} = 0.40049, ϕ_{37} = -0.31171
EC complex	ϕ_{38} = 0.80816, ϕ_{39} = -0.37854
RVE8 mRNA	ϕ_{40} = 2.2417, ϕ_{41} = 1.4074, ϕ_{42} = -1.5159
RVE8 protein	ϕ_{43} = 0.32848, ϕ_{44} = -0.14877
ELF3 mRNA	ϕ_{45} = 0.014607, ϕ_{46} = 0.23392, ϕ_{47} = -0.27265
ELF3 protein	ϕ_{48} = 14.0123, ϕ_{49} = -17.3569
ELF4 mRNA	ϕ_{50} = 0.014384, ϕ_{51} = 0.032117, ϕ_{52} = 0.051737
	ϕ_{53} = -11.007
ELF4 protein	ϕ_{54} = 0.67365, ϕ_{55} = -0.39718

S4 Table. (Continued)

mRNA/Protein	Parameter value
LUX mRNA	ϕ_{56} = 0.028907, ϕ_{57} = 0.01727, ϕ_{58} = 0.034063
	ϕ_{59} = -26.4166
LUX protein	ϕ_{60} = 2.461, ϕ_{61} = -1.4794
GI mRNA	ϕ_{62} = 0.0002445, ϕ_{63} = 0.072434
	ϕ_{64} = 0.11811, ϕ_{65} = 0.456, ϕ_{66} =-0.58448
GI protein	ϕ_{67} = 0.40246, ϕ_{68} = -0.78572, ϕ_{69} = -0.020811
COP1 protein	ϕ_{70} = -0.80938, ϕ_{71} = -0.8102, ϕ_{72} = 0.91424
ZTL protein	ϕ_{73} = 2.3354, ϕ_{74} = -0.44539,ϕ_{75} = 0.027925
