# Supplementary material for: Kernel Architecture of the Genetic Circuitry of the Arabidopsis Circadian System
Source: PLoS Comput Biol. 2016 Feb 1;12(2):e1004748. doi: 10.1371/journal.pcbi.1004748 (PMC4734688; doi:10.1371/journal.pcbi.1004748)
Supplement: S5 Table — (PDF) [file pcbi.1004748.s017.pdf]

S5 Table. Re-optimized parameters of loops I to IV. Refer to S1 Text.

Loop	mRNA/Protein	Parameter value
I	Protein P	α_1 = -0.43301, α_2 = -0.4949, α_3 = 0.4358
	LHY mRNA	α_4 = 4.8391, α_5 = 2.9317, α_6 = 8.9448, α_7 = -0.97069
	LHY protein	α_8 = 0.91961, α_9 = -0.69932
	PRR9 mRNA	α_{10} = 0.0014509, α_{11} = -0.50361
	PRR9 protein	α_{12} = 0.075863, α_{13} = -1.9014
	PRR5 mRNA	α_{14} = 0.015717, α_{15} = 0.10754, α_{16} = 0.052008, α_{17} = -52.317
	PRR5 protein	α_{18} = 5.5115, α_{19} = -2.9011
	TOC1 mRNA	α_{20} = 0.045856, α_{21} = 0.15729, α_{22} = -3.5101
	TOC1 protein	α_{23} = 0.61764, α_{24} = -0.46954
II	Protein P	β_1 = -0.43301, β_2 = -0.4949, β_3 = 0.4358
	LHY mRNA	β_4 = 1.6849, β_5 = 1.0514, β_6 = 0.56448, β_7 = -1.1406
	LHY protein	β_8 = 0.63159, β_9 = -0.46862
	PRR9 mRNA	β_{10} = 0.0026547, β_{11} = -0.60063
	PRR9 protein	β_{12} = 0.051438, β_{13} = -1.7146
	PRR7 mRNA	β_{14} = 0.0033064, β_{15} = 0.10306, β_{16} = -0.29026
	PRR7 protein	β_{17} = 0.37496, β_{18} = -0.25051
	TOC1 mRNA	β_{19} = 0.036621, β_{20} = 0.089066, β_{21} = -4.5098
	TOC1 protein	β_{22} = 0.47306, β_{23} = -0.34138

S5 Table. (Continued)

Loop	mRNA/Protein	Parameter value
III	Protein P	γ_1 = -0.43301, γ_2 = -0.4949,
		γ_3 = 0.4358
	LHY mRNA	γ_4 = 0.032732, γ_5 = 0.11689,
		γ_6 = 0.34663, γ_7 = -1.4559
	LHY protein	γ_8 = 0.73725, γ_9 = -0.45881
	PRR9 mRNA	γ_{10} = 0.39135, γ_{11} = -0.14337
	PRR9 protein	γ_{12} = 1.3024, γ_{13} = -0.35734
	PRR7 mRNA	γ_{14} = 0.0067299, γ_{15} = 0.00039876,
		γ_{16} = -0.35087
	PRR7 protein	γ_{17} = 0.33168, γ_{18} = -0.2004
	EC complex	γ_{19} = 0.69819, γ_{20} = -0.41231
	ELF3 mRNA	γ_{21} = 0.076138, γ_{22} = 0.4676,
		γ_{23} = -0.44998
	ELF3 protein	γ_{24} = 1.0179, γ_{25} = -2.1412
	ELF4 mRNA	γ_{26} = 0.027705, γ_{27} = 0.091673,
		γ_{28} = 0.087689, γ_{29} = -4.3341
	ELF4 protein	γ_{30} = 1.7948, γ_{31} = -2.2835
	LUX mRNA	γ_{32} = 0.028849, γ_{33} = 0.10797,
		γ_{34} = 0.00088557, γ_{35} = -5.1109
	LUX protein	γ_{36} = 1.9206, γ_{37} = -0.46888
IV	Protein P	η_1 = -0.43301, η_2 = -0.4949,
		η_3 = 0.4358
	LHY mRNA	η_4 = 1.4534e-8, η_5 = 1.92858e-5,
		η_6 = 2.1802e-5, η_7 = -0.31409
	LHY protein	η_8 = 0.3093, η_9 = -0.27172
	PRR9 mRNA	η_{10} = 0.0062145, η_{11} = 0.35073,
		η_{12} = 1.3424, η_{13} = -0.29322
	PRR9 protein	η_{14} = 1.0058, η_{15} = -0.25442
